# Unveiling host–guest–solvent interactions in solution by identifying highly unstable host–guest configurations in thermal non-equilibrium gas phase

**DOI:** 10.1038/s41598-022-12226-0

**Published:** 2022-05-17

**Authors:** Hyoju Choi, Young-Ho Oh, Soojin Park, Sung-Sik Lee, Han Bin Oh, Sungyul Lee

**Affiliations:** 1 Department of Applied Chemistry, Kyung Hee University, Gyeonggi, 17104 Republic of Korea; 2 Department of Chemistry, Sogang University, Seoul, 121-742 Republic of Korea

**Keywords:** Materials chemistry, Physical chemistry, Supramolecular chemistry, Biophysics, Chemistry

## Abstract

We propose a novel scheme of examining the host–guest–solvent interactions in solution from their gas phase structures. By adopting the permethylated β-cyclodextrin (perm β-CD)–protonated L-Lysine non-covalent complex as a prototypical system, we present the infrared multiple photon dissociation (IRMPD) spectrum of the *gas phase* complex produced by electrospray ionization technique. In order to elucidate the structure of perm β-CD)/LysH^+^ complex in the gas phase, we carry out quantum chemical calculations to assign the two strong peaks at 3,340 and 3,560 cm^−1^ in the IRMPD spectrum, finding that the carboxyl forms loose hydrogen bonding with the perm β-CD, whereas the ammonium group of L-Lysine is away from the perm β-CD unit. By simulating the structures of perm β-CD/H^+^/L-Lysine complex *in solution* using the supramolecule/continuum model, we find that the extremely unstable gas phase structure corresponds to the most stable conformer in solution.

## Introduction

Host–guest interactions are of fundamental importance as the principle of molecular recognition and self-assembly^1–3^. “Bottom-up” construction of supramolecular functional materials^4–7^ based on host–guest interactions has found wide applications for catalysts (artificial enzymes)^8,9^, sensing^10^, drug delivery^11,12^, gene delivery^13,14^, bioimaging^15,16^, stimuli-responsive materials^17^ and photodynamic therapy^18,19^. Examining the host–guest configurations has been the focus of many intensive studies^20–22^, however, scrutinizing them in solution phase at molecular level is usually a difficult task to achieve. The two components of host–guest complexes are held together with highly specific binding by non-covalent forces, most commonly by hydrogen bonding. The host components are usually large molecules such as cyclodextrins^23,24^, calix[n]arenes^25,26^, or cucurbit[n]urils^27^. The hydrophobic cavity of a macrocyclic host molecule usually encapsulates some part of the guest, whereas the outer rims of the host tend to be hydrophilic. Details for the interactions and bindings between the functional groups in the host and guest are of specific interest in relation to molecular recognition. One of the most intriguing questions concerning the host–guest complexes would be whether their structures observed in gas-phase may reflect the host–guest–solvent interactions in solution or not. If there exists a close relationship between the structures of host–guest complexes in the two phases, then determining their configurations in the gas phase, which might be more unambiguous than the solution phase structures (at least due to the solvent-free environment) may give invaluable information for the detailed interactions between the host and guest (and also solvent molecules). By addressing this highly critical question, we describe here a novel protocol for examining the host–guest–solvent interactions in *solution* by elucidating the structures of *gas phase* host–guest complexes. We employ the perm β-CD/LysH^+^ complex as a prototypical host–guest system to advance the scheme.

First, we obtain the IRMPD spectra of the gas phase perm β-CD/H^+^/L-Lys complex produced by the ESI/MS procedure. We determine the gas phase structure of perm β-CD/H^+^/L-Lys by comparing the calculated IR spectra with the experimentally observed IRMPD^28–39^ spectrum of the perm-CD/ L-LysH^+^ complex. We carry out quantum chemical calculations for the two intense bands at 3340 and 3560 cm^−1^, assigning them to stretch modes of the side chain ammonium and the carboxyl group, respectively. Second, we show that Gibbs free energy of the experimentally observed gas phase complex (Complex I) obtained by ESI/mass spectrometry (ESI/MS) from solution phase is much higher (by ~ 30 kcal/mol) than that of the lower Gibbs free energy structure (Complex II), demonstrating that the gas phase complex is highly unstable in thermodynamic sense (as was also observed for gas phase perm β-CD/H^+^/G, G = alanine^40^, isoleucine^41^, H_2_O^42^). Third, by adopting the supramolecule/continuum approach, we simulate the structures of perm β-CD/H^+^/L-Lys complex in solution, thereby finding that the relative thermodynamic stability of perm β-CD/H^+^/L-Lys complex in solution is the complete *reversal* of that in the gas phase: The extremely higher Gibbs free energy gas phase structure (Complex I) corresponds to the most stable conformer (Complex A) in solution, and the most stable gas phase conformer (Complex II) becomes a less stable one (Complex B) in solution. This clearly indicates that the origin of the high Gibbs free energy *gas phase* structure (Complex I) is the configuration (Complex A) of perm β-CD/H^+^/L-Lys in *solution*, suggesting that the structure of gas phase host–guest complexes produced by ESI/MS/IRMPD technique^43^ may be used as a very useful and accurate guide for probing host–guest–solvent interaction in solution.

## Results

### IRMPD spectrum and structure of thermodynamically highly unstable gas phase host–guest complex

**Figure 1:**
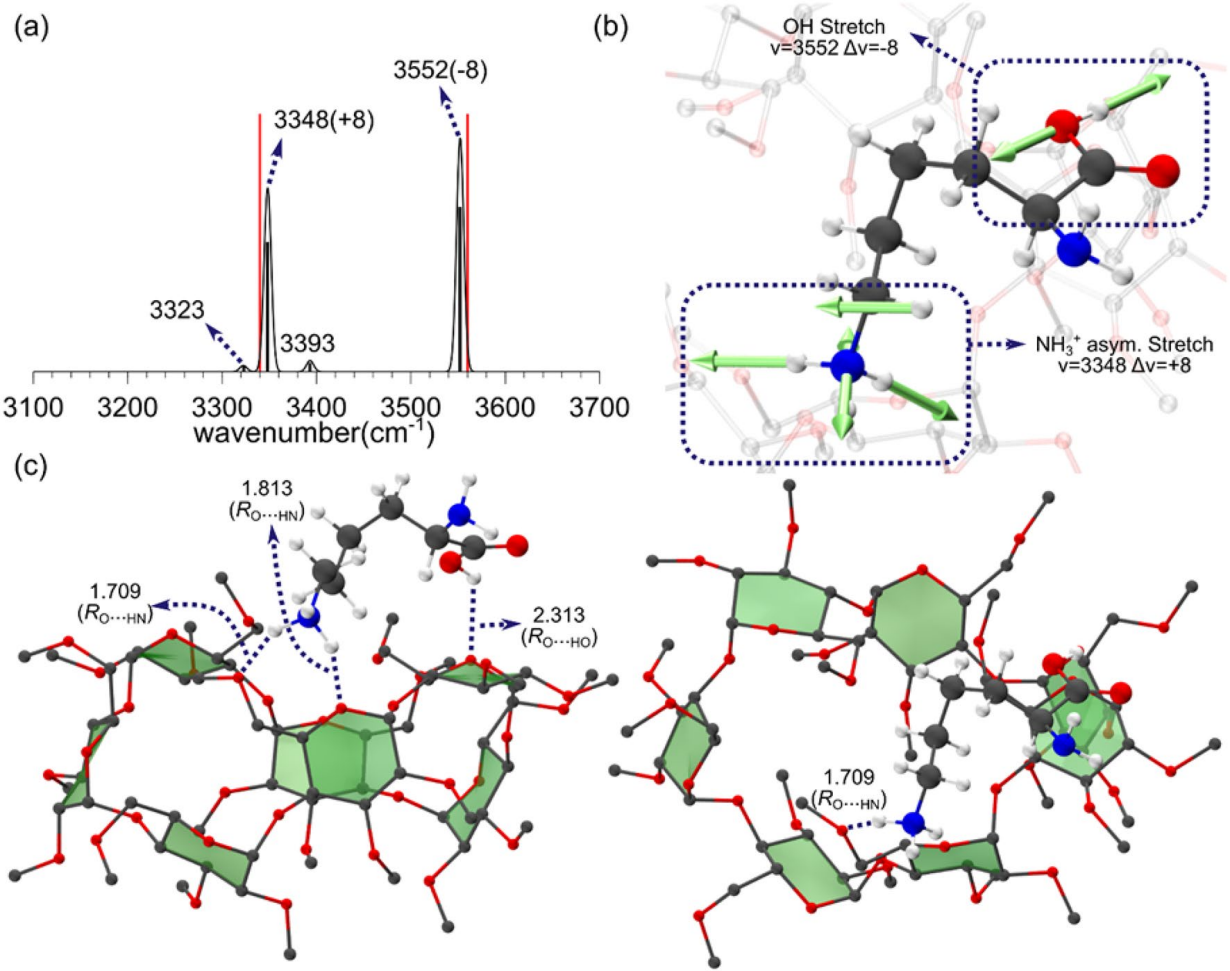
(**a**) Theoretical IR spectrum, (**b**) normal modes, and (**c**) two views of the structure of Complex I of gas phase perm β-CD–protonated L-Lys. Red sticks represent the positions of experimental IRMPD bands. Hydrogen atoms in permetylated β-cyclodextrin are omitted for clarity. Red: oxygen, blue: nitrogen, gray: carbon, white: hydrogen.

**Figure 2:**
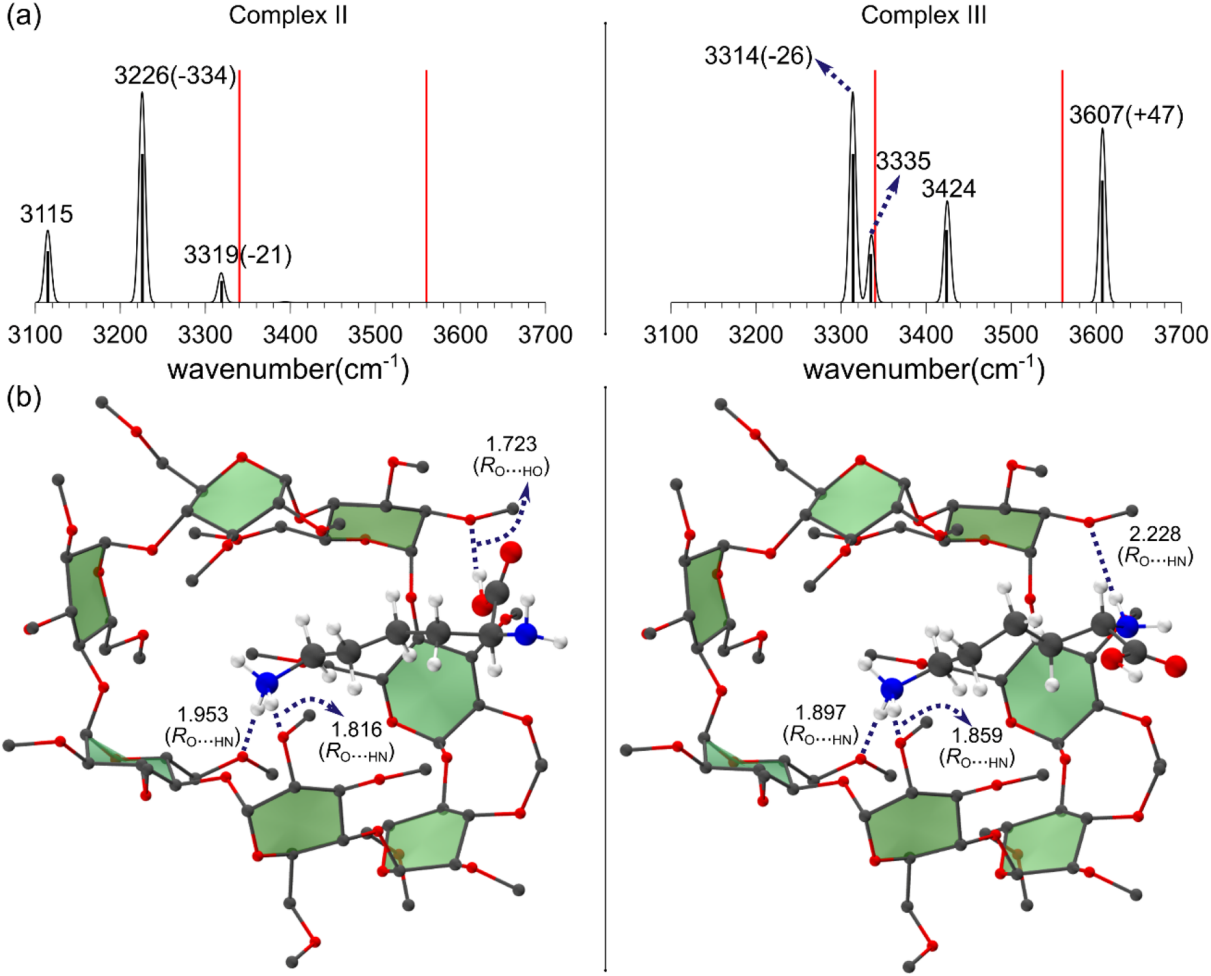
(**a**) Theoretical IR spectra and (**b**) structures of lower Gibbs free energy perm β-CD–protonated L-Lys complexes II and III in gas phase.

Figure 1a presents the experimental IRMPD spectrum of gas phase perm β-CD–protonated L-Lys complex in the region > 3100 cm^−1^. Two intense bands at 3340 and 3560 cm^−1^ are noticeable. The band at 3560 cm^−1^ is very similar to carboxyl stretch band (~ 3570 cm^−1^) of the “free” gas phase amino acids^44^, indicating that it may be assigned a –OH stretch mode of nearly isolated carbonyl group. The theoretical spectrum (calculated at wB97X-D/6-31G* level of theory, using a scale factor of 0.940868 to correct for the DFT errors and anharmonic effects) of Complex I, which fully accounts for the IRMPD spectrum, is also depicted in Fig. 1a along with the normal modes of the absorption bands in Fig. 1b, and the corresponding gas phase perm β-CD/H^+^/L-Lys structure. The calculated infrared (IR) spectrum for Complex I exhibits two strong bands at 3348 and 3552 cm^−1^, which is the characteristic feature of the experimental spectrum in Fig. 1a. The two intense bands at 3226 and 3319 cm^−1^ in the IR spectrum are assigned as the asymmetric stretch of the ammonium and the carboxyl –OH stretch mode, respectively (Fig. 2a). Very weak absorptions at 3393 and at 3323 cm^−1^ are asymmetric and symmetric –NH_2_ stretch modes, respectively, but they seem to be so weak that they are almost indistinguishable from background noise in the experimental spectrum.

The presence or absence of hydrogen bonding between the perm β-CD unit and the functional groups (carbonyl, amino, and ammonium), and their locations with respect to the host perm β-CD are the critical features in the gas phase structures of the complexes. In Complex I (see Fig. 1c) the amino and the side chain ammonium groups are far off the perm β-CD unit, whereas the carboxyl loosely interact with the O atoms of the perm β-CD. The weak interactions (*R*_OH_ = 2.313 Å) between the carboxyl and the perm β-CD unit was also seen in the structure of gas phase perm β-CD/H^+^/Isoleucine^41^ system (with *R*_O_.._HO_ = 2.053 Å). The calculated length of hydrogen bonding (*R*_O_…_HO_ = 2.313 Å), which is much larger than that observed for ordinary hydrogen bonding (~ 1.70 Å), indicates that interactions between the carboxyl and the perm β-CD unit are quite weak in Complex I, in agreement with the high frequency –OH stretch band at 3552 cm^−1^ in IRMPD spectrum. The origin of this intriguing loose hydrogen bonding of the carboxyl to the perm β-CD can be accounted for by envisaging an intervening water molecule in solution phase, as discussed below.

### Gas phase complexes of much lower Gibbs free energies

Of the thermodynamically more stable (lower Gibbs free energy) conformers of gas phase perm β-CD/H^+^/L-Lys complexes, Fig. 2 presents the two structures (Complex II, III) deserving close inspection. In Complex II and Complex III the ammonium is encapsulated inside the perm β-CD unit, with the carboxyl and the amino group interacting with the host at its rim, respectively. Figure 2 also shows that the calculated IR frequency of carbonyl –OH stretch in Complex II is enormously *red*-shifted to 3226 cm^−1^ (the corresponding normal mode is depicted in Supplementary Information (Figure S1)), as the result of strong hydrogen bonding (*R*_O_…_HO_ = 1.723 Å, considerably shorter than *R*_O_…_HO_ = 2.313 Å in Complex I) between –CO_2_H and the perm β-CD unit. On the other hand, the corresponding frequency in Complex III is calculated to be *blue*-shifted (Fig. 2b) from the experimental band to 3607 cm^−1^. This indicates that in Complex III the carboxyl is completely *isolated*, free from the influence of the perm β-CD ring, as can be seen from its structure in Fig. 2 (–CO_2_H is bent outward away from the perm β-CD unit). In this latter conformer, the α–amino group forms a weak hydrogen bond with the CD O atoms (*R*_O_…_HN_ = 2.228 Å), giving the high frequency symmetric and asymmetric –NH_2_ stretch modes at 3335 and 3424 cm^−1^, respectively. It should be noted that the calculated IR spectra for Complex II and Complex III are in complete disagreement with the experimental IRMPD spectrum in Fig. 1a.

Table 1 lists the relative Gibbs free energies of the gas phase complexes I–III. We find that Complex II and III is much more stable (lower Gibbs free energy) than other complexes by 27–30 kcal/mol. Two structural differences are to be noted between Complex I and Complex II/III): First, in Complex I interactions between the amino and carboxyl with the perm β-CD unit is much weaker than in Complex II/III. Second, in the latter two complexes the side chain –NH_3_^+^ is encapsulated inside the cavity of the host perm β-CD, whereas it is essentially isolated in Complex I. Thus, it seems that these stronger interactions between the LysH^+^ functional groups and the perm β-CD provide Complex II and III far greater thermodynamic stability than Complex I.

**Table 1:** Thermodynamic properties of permethylated β-CD–protonated L-Lys complexes in gas phase ((I)–(III)) and in solution ((A), (B), (C)).

	Gas phase			In solution		
	(I)	(II)	(III)	(A)	(B)	(C)
*E*^a^	− 5597.93965	− 5597.98887	− 5597.98501	− 6819.34126^f^ − 6821.02509^g^	− 6819.33577^f^ − 6821.02012^g^	− 6819.32416^f^ − 6821.01128^g^
*ΔE*^b^	30.9	0	2.4	0	3.4^f^ 3.1^g^	10.7^f^ 8.7^g^
*B. E*	− 27.7	− 78.6	− 76.0	–	–	–
*G*^d^	− 5597.76249	− 5597.81018	− 5597.80576	− 6818.79107^f^	− 6818.78672^f^	− 6818.77524^f^
*ΔG*_298K_^e^	29.9	0	2.8	0	2.7^f^	9.9^f^

^a^Electronic energy in Hartree, wB97X-D/6-311G**.

^b^Relative electronic energy in kcal/mol, wB97X-D/6-311G**.

^c^Gas phase binding energy (*H*_complex_–*H*_host_–*H*_guest_) in kcal/mol.

^d^Gibbs free energy at 298 K in Hartree.

^e^Relative Gibbs free energy in kcal/mol.

^f^wB97X-D/6-31G**; ^g^wB97X-D/6-311G** //wB97X-D/6-31G**.

From these findings, it is clear that thermal equilibrium has *not* been achieved for the gas phase perm β-CD/L-LysH^+^ complexes. The reason for the striking observation of thermodynamically highly unstable conformer (Complex I), not the thermally much more stable conformers (Complex II, III), is extremely intriguing. One plausible proposition is that when a mixture of perm β-CD and L-LysH^+^ in solution was being transferred into the gas phase, the complex may not fully relax to the global minimum, kinetically trapped in the local minimum (Complex I). This situation may correspond to the case in which the thermodynamically much less stable (higher Gibbs free energy) gas phase conformer (Complex I) may actually be the most stable configuration of perm β-CD/L-LysH^+^ complex in *solution* phase under the influence of solvating molecules, and thus the solution phase perm β-CD/L-LysH^+^ complex that is structurally close to Complex I is observed in the gas phase before thermal equilibrium is attained.

### The most stable configuration (Complex A) in solution phase as the origin of the high Gibbs free energy gas phase host–guest conformer (Complex I)

**Figure 3:**
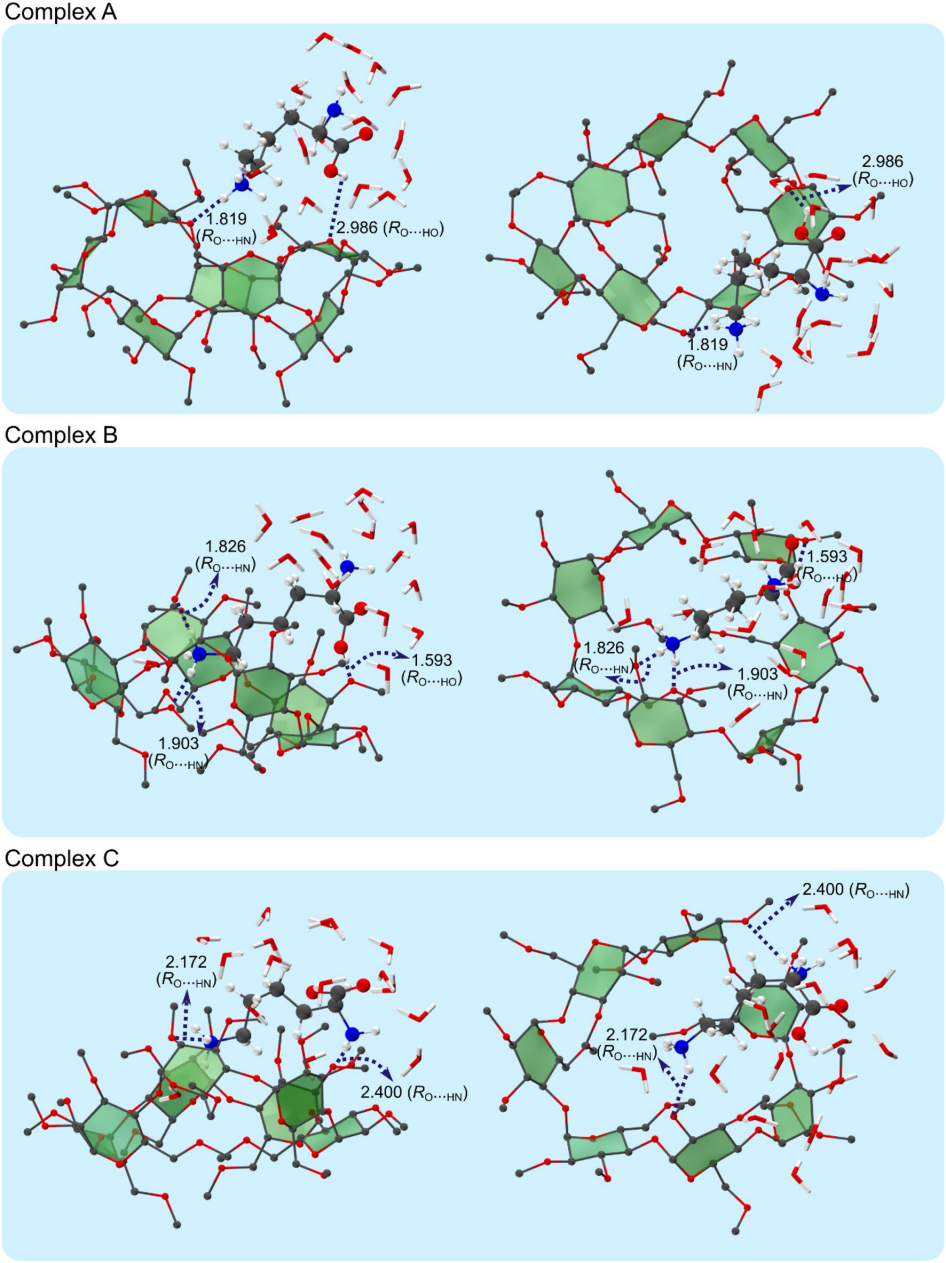
Two views of the structures (Complex A, B and C) of perm β-CD–protonated L-Lys complexes in solution, based on the gas phase (**a**) Complex I, (**b**) Complex II, and (**c**) Complex III, respectively. Pale blue background represents solvent continuum.

**Figure 4:**
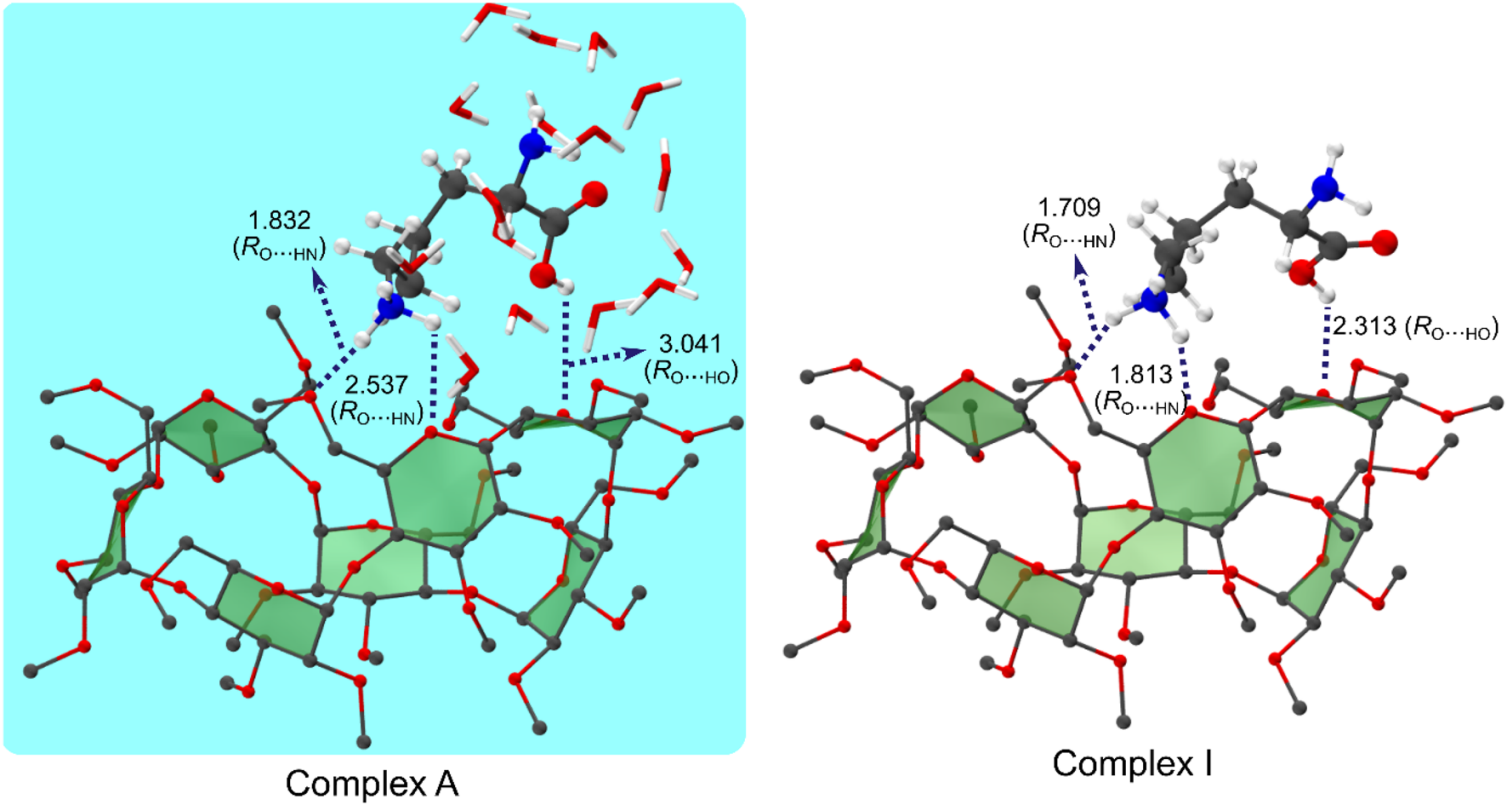
Comparison of perm-CD–LysH^+^ configurations in solution (Complex A, wB97X-D/6-31G*) and in gas phase (Complex I, wB97X-D/6-311G*).

To unravel the origin of the extremely high Gibbs free energy perm β-CD/L-LysH^+^ non-covalent complex in gas phase, we adopt a model of solution phase host–guest system by employing the supramolecule/continuum approach, by which we treat the solvent in the first shell around the complex as explicit molecules, and other numerous solvent molecules in the second shell and beyond as continuum. By allowing the gas phase complexes (Complex I, II and III) to interact with increasing number of explicit H_2_O molecules, we find that the model including 16 explicit H_2_O molecules fully surrounding the β-CD/L-LysH^+^ complex in the first shell plus water continuum is the best one with regard to the amounts of computational efforts. Figure 3 and Table 1 present the lowest Gibbs free energy solution phase structures (Complex A, B and C) corresponding to the three gas phase conformers (Complex I, II and III, respectively) obtained by extensive search over the potential landscape of the perm β-CD/L-LysH^+^ system in solution. (Some other low-lying (local minimum Gibbs free energy) structures of are given in Figure S2) We find that Complex A, conforming to the highly unstable gas phase Complex I, is now calculated to be *more stable* (Gibbs free energy *lower* by 2.73 at wB97X-D/6-31G*) than Complex B corresponding to the most stable gas phase Complex II, exhibiting a complete *reversal* of their relative thermodynamic stability in the gas phase by overcoming the huge deficit of ~ 30 kcal/mol. The Gibbs free energy of the solution phase structure C stemming from the gas phase structure III is calculated to be much higher (> 9.9 kcal/mol). This seems to result from the differences in the structural features of Complex A vs. Complex B/C: In all three complexes the carboxyl interacts both with a water molecule and the perm β-CD unit, but the strength of interactions decreases from A to B to C, the H_2_O–perm β-CD distance increasing from 1.601 to 2.075 and to 2.432 Å. Moreover, two water molecules directly interact with the side chain –NH_3_^+^, and are in close contact with other H_2_O molecules in a chain-like form in Complex A. In contrast, only a more or less isolated H_2_O molecule binds to the latter functional group inside the cavity of the perm β-CD unit in Complex B/C. Therefore, we conclude that the most stable Complex A in solution is effused by the ESI method into the gas phase, and that the resulting Complex I is observed in gas phase without thermal relaxation to the lower free energy gas phase structures. From the structure of Complex A in Fig. 3, we may now visualize the host–guest–solvent interactions between the perm β-CD, L-LysH^+^ and solvent molecules in solution: The L-LysH^+^ guest situates at the rim of the perm-CD host, not encapsulated inside it. The ammonium forms weak hydrogen bonding with the perm β-CD unit, being surrounded by two water molecules. The amino group is enveloped by 3 H_2_O molecules off the host perm β-CD. The carboxyl weakly interacts with the perm β-CD bridged by a water molecule, also binding to 3 H_2_O molecules away from the host. These solvating water molecules are linked by a number of other interconnecting water molecules, together forming the first shell around the perm β-CD/L-LysH^+^ complex. (Details of interactions between the perm β-CD host, the L-LysH^+^ guest and solvent molecules in solution are described in Figure S3–S5). It would also be worth noticing that the structural features (locations of the guest functional groups relative to the host) of the host–guest complex in solution phase (Complex A) are more or less retained in gas phase (Complex I), although some bond distances seem to relax closer to the host when the solvent molecules are removed from Complex A to form the gas phase Complex I by the ESI procedure: For example, the distance between an ammonium H atom and a perm β-CD O atom in Complex A becomes shorter (from 2.537 to 1.813 Å), and that between the carboxyl H atom and a perm β-CD O atom relaxes from 3.041 to 2.313 Å (Fig. 4).

### Elucidating the host–guest–solvent interactions in solution phase from the gas phase host–guest configurations

In summary, our present study gives an unambiguous clue to the origin of the high Gibbs free energy perm β-CD/H^+^/Ala, Ile, L-Lys and H_2_O non-covalent complexes experimentally observed in gas phase: Their source is the perm β-CD/guest complexes in solution from which the gas phase complexes are produced by the ESI/MS before reaching thermal relaxation to equilibrium. The IRMPD and quantum chemical methods were employed here to determine the structures of the gas phase host–guest complexes, but any other techniques may also be useful. These findings offer a profound implication and a novel scheme with a view to solution phase configurations of host–guest complexes: The structures of the gas phase host–guest complexes produced and identified by the ESI/IRMPD spectroscopy carry important and accurate information for their configurations in solution phase. That is, by “freezing” the host–guest complexes and determining their structures in the gas phase, it is possible to ‘fingerprint’ the host–guest–solvent configurations in solution.

## Methods

### Experimental: Infrared multiple photon dissociation (IRMPD) spectroscopy

Infrared multiple photon dissociation spectroscopy experiments^28–39^ were performed on a 4.7 T ESI-Fourier transform ion cyclotron resonance (FT-ICR) mass spectrometer (Ionspec, Lake Forest, CA, USA). The detailed IRMPD spectroscopy setup was described in detail elsewhere^42^. Non-covalent complexes of permethylated β-CD , protonated L-Lys and water, *i.e*., (β-CD + L-Lys + H)^+^, were generated by electrospraying a solution of 20 mM permethylated β-CD and L-Lys in 49:49:2 (v/v/v) H_2_O:MeOH:AcOH. A permethylated β-CD was chosen as a main target of study instead of native β-CD in order to simplify the IR spectrum, and thus to facilitate the interpretation of the IR spectrum. Non-covalent complexes were isolated in the ICR cell using stored waveform inverse Fourier transform (SWIFT) waveforms and stored in the ICR cell for 25 s. The stored non-covalent complexes were irradiated using IR laser light pulses (10 Hz). A photodissociation spectrum was obtained by recording the abundance of the (β-CD + L-Lys + H^+^) peak with (A) and without (A_0_) infrared laser irradiation as a function of the irradiated laser wavenumber in cm^−1^; ion-depletion measurement. The abundance of the precursor non-covalent complex ion was monitored in 5 nm steps. The abundance (A and A_0_) of the precursor complex ion was read directly from the data acquisition software without any data processing such as apodization or zero-filling.

An IRMPD spectrum was obtained in the wavenumber region from 3050 to 3750 cm^−1^, using a commercial OPO laser (single stage optical parametric oscillator with an unseeded cavity design and a bulk potassium titanyl arsenate (KTA) crystal, IROPO, OPOTek, Carlsbad, CA, USA). For the IRMPD spectrum, the recorded depletion of precursor complex ions was normalized using the measured (and smoothed) laser power assuming a linear dependence. The IR laser irradiation time was adjusted using a mechanical shutter (UNIBLITZ, Vincent Associates, Rochester, NY, USA) that was synchronized with an FT-ICR TTL signal with some delay time. The IR laser light was collimated using a series of irises with adjustable apertures and was carefully aligned to maximize the overlap between ions in the ICR cell and the laser light. The IR laser was focused using a CaF_2_ lens with a 1 m focal length.

Permethylated β-CD was purchased from Sejin Chemical Industry Co. (Seoul, Korea) and was used without purification. Water and methanol were of HPLC grade (Fischer, Seoul, Korea), acetic acid and L-Lys were purchased from Sigma (Seoul, Korea).

### Modelling and computational details

All calculations were conducted using the wB97X-D functional, which may treat weak interactions (hydrogen bonding included) very well, with 6-311G** and 6-31G** basis set implemented in Gaussian09 suite of programs^45^. For the structures in solution we also carried out single point calculations using the wB97X-D/6-311G** method at the wB97X-D/6-31G** optimized geometry. We obtained the conformers of gas phase perm β-CD/H^+^/L-Lys based on the structures of the perm β-CD unit described in our previous work^42^, wherein the NVT ensemble molecular dynamics simulations and PM6 calculations were performed for the gas phase complexes, using the energy window of 35.4 kcal/mol for the lowest 30 conformers. A scale factor of 0.940868 was used to best fit the calculated IR frequencies to experimental spectrum by the least square procedure. All structures were obtained by verifying all real vibrational frequencies. For the corresponding structures of perm β-CD/LysH^+^ complex in solution, we used the supramolecule/continuum approach, in which the solvent molecules directly interacting with –CO_2_H, -NH_2_ and side chain ammonium groups in the first shell around the complex are treated as explicit molecules, and other numerous H_2_O molecules in the second shell and beyond as water continuum by the SMD method^46^. Although we used as solvent the mixture of water, methanol, and acetic acid (49:49:2 volume ratio), we modelled our present system as the perm β-CD/LysH^+^ complex in water for simplicity and by invoking two presumptions: First, methanol would preferentially evaporate as the ESI droplets becomes smaller due to higher volatility. Second, H_2_O molecules would mostly constitute the first shell around the perm β-CD/LysH^+^ complex because the two -OH groups per H_2_O molecule would form much stronger hydrogen bonding with the functional groups (ammonium, amino and carboxyl) than CH_3_OH would. We proceeded by adding water molecules to the functional groups in perm β-CD/H^+^/L-Lys complex, beginning with 4 H_2_O molecules, searching for the lowest Gibbs free energy configurations at each step. The Gibbs free energies were calculated by the standard options (bulk electrostatic contribution from a self-consistent reaction field treatment by superposition of nuclear-centered spheres, and short-range interactions between the solute and solvent molecules in the first shell obtained by geometry-dependent atomic surface tensions, etc.) for the keyword SCRF = (SMD, solvent = water). Comparisons are made with those optimized from initial configurations with by randomly added water molecules using the VMD (visual molecular dynamics) program.

## Supplementary Information

Supplementary Information.

## Data Availability

The datasets generated and/or analysed during the current study are available in the Computational Chemistry Results Repository (https://www.iochem-bd.org/), 10.19061/iochem-bd-6-124.
