# Supplementary material for: Unveiling host–guest–solvent interactions in solution by identifying highly unstable host–guest configurations in thermal non-equilibrium gas phase
Source: Sci Rep. 2022 May 17;12:8169. doi: 10.1038/s41598-022-12226-0 (PMC9114120; doi:10.1038/s41598-022-12226-0)
Supplement: Supplementary file 1 — Supplementary Information. [file 41598_2022_12226_MOESM1_ESM.docx]

**Supplementary Information**

**Unveiling Host-Guest-Solvent Interactions in Solution by Identifying Highly Unstable Host-Guest Configurations in Thermal Non-Equilibrium Gas Phase**

Hyoju Choi,^1^† Young-Ho Oh,^1^† Soojin Park,^2^ Sung-Sik Lee,^1^ Han Bin Oh^2*^, Sungyul Lee^1*^

^1^Department of Applied Chemistry, Kyung Hee University; Gyeonggi 17104, Republic of

Korea

^2^Department of Chemistry, Sogang University; Seoul 121-742, Republic of Korea

Fig. S1.


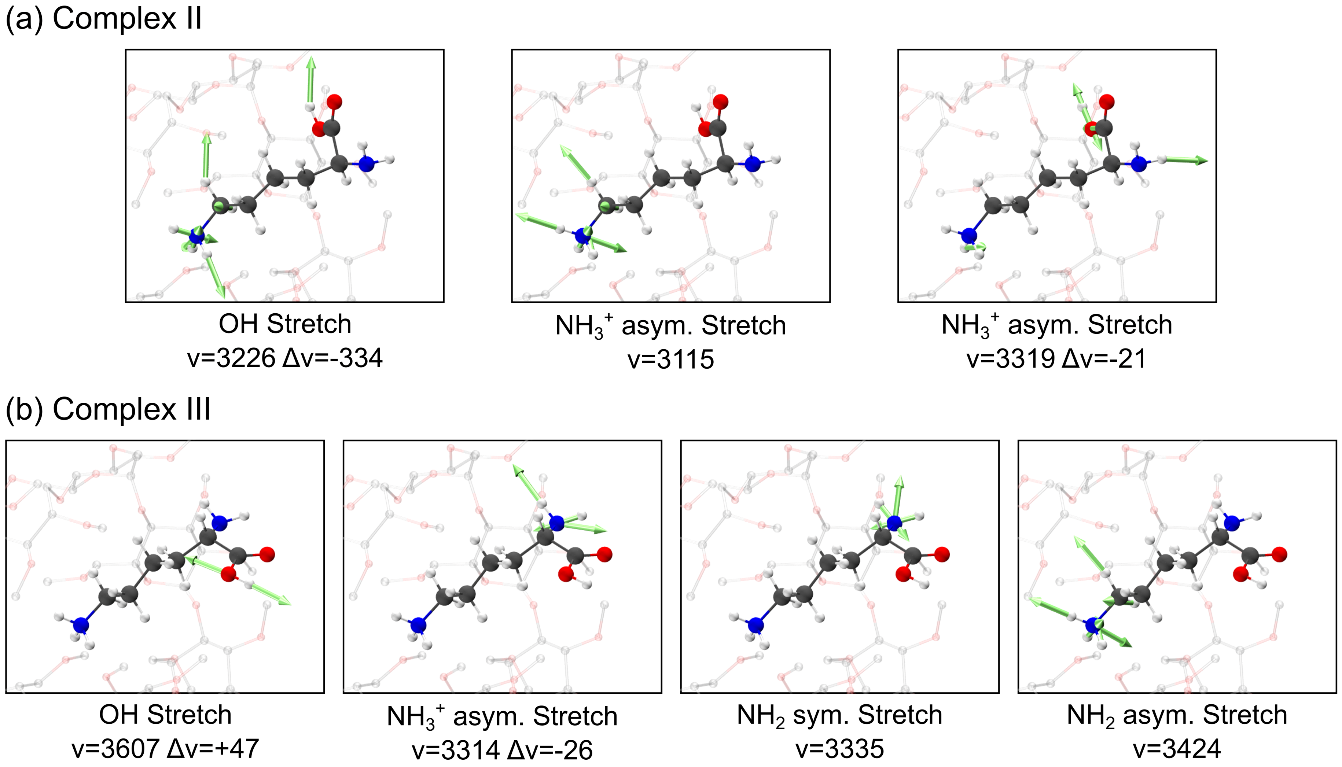


Figure S1. Normal modes and harmonic frequencies (cm^-1^) of gas phase (a) Complex II and (b) Complex III. Δν denotes the deviations from the experimental IRMPD bands. Red: oxygen, blue: nitrogen, gray: carbon, white: hydrogen.

Fig. S2.


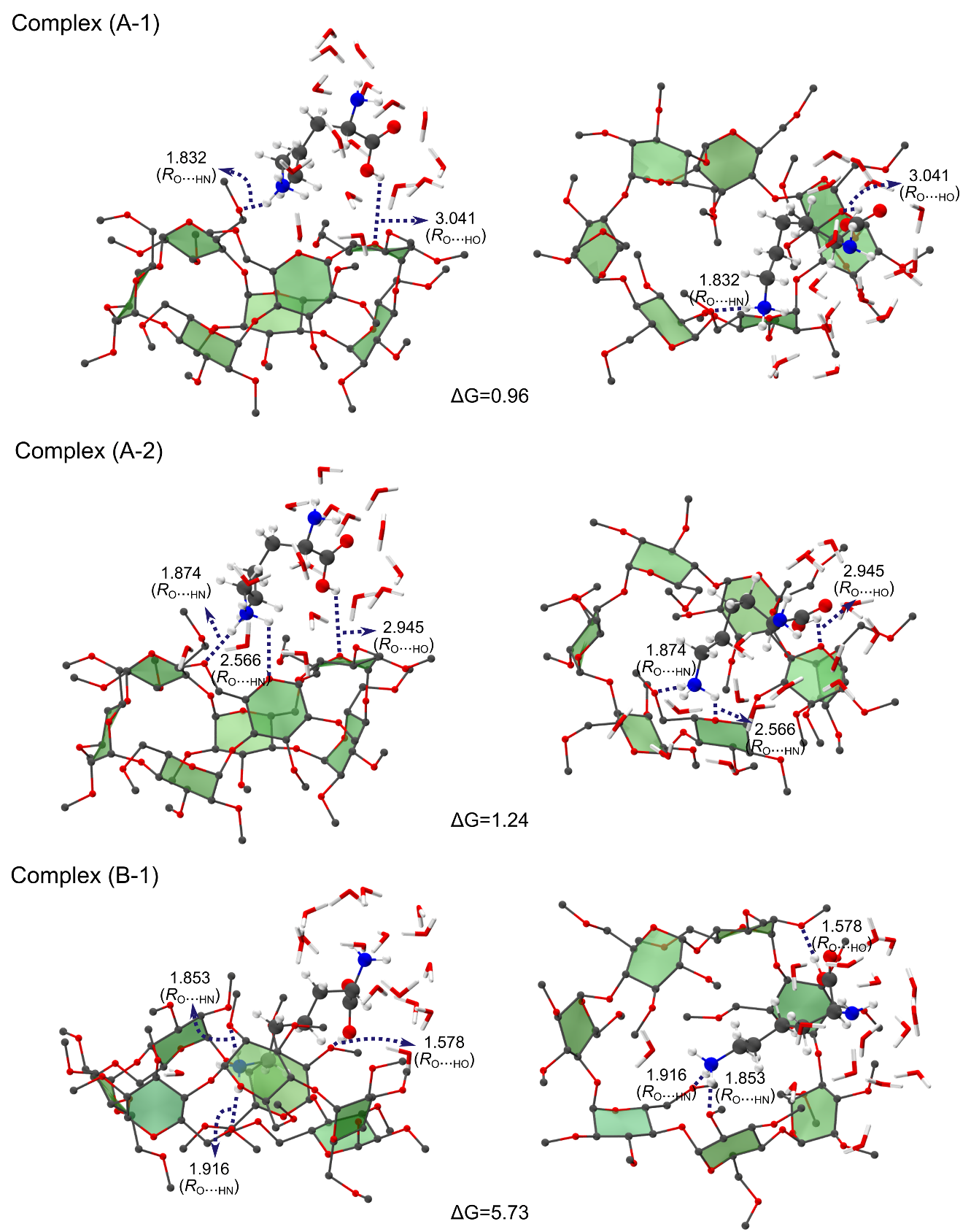


Figure S2. Structures of some low-lying perm-CD/LysH^+^ complexes in solution. Relative Gibbs free energy with respect to Complex A in kcal/mol.

Fig. S3.


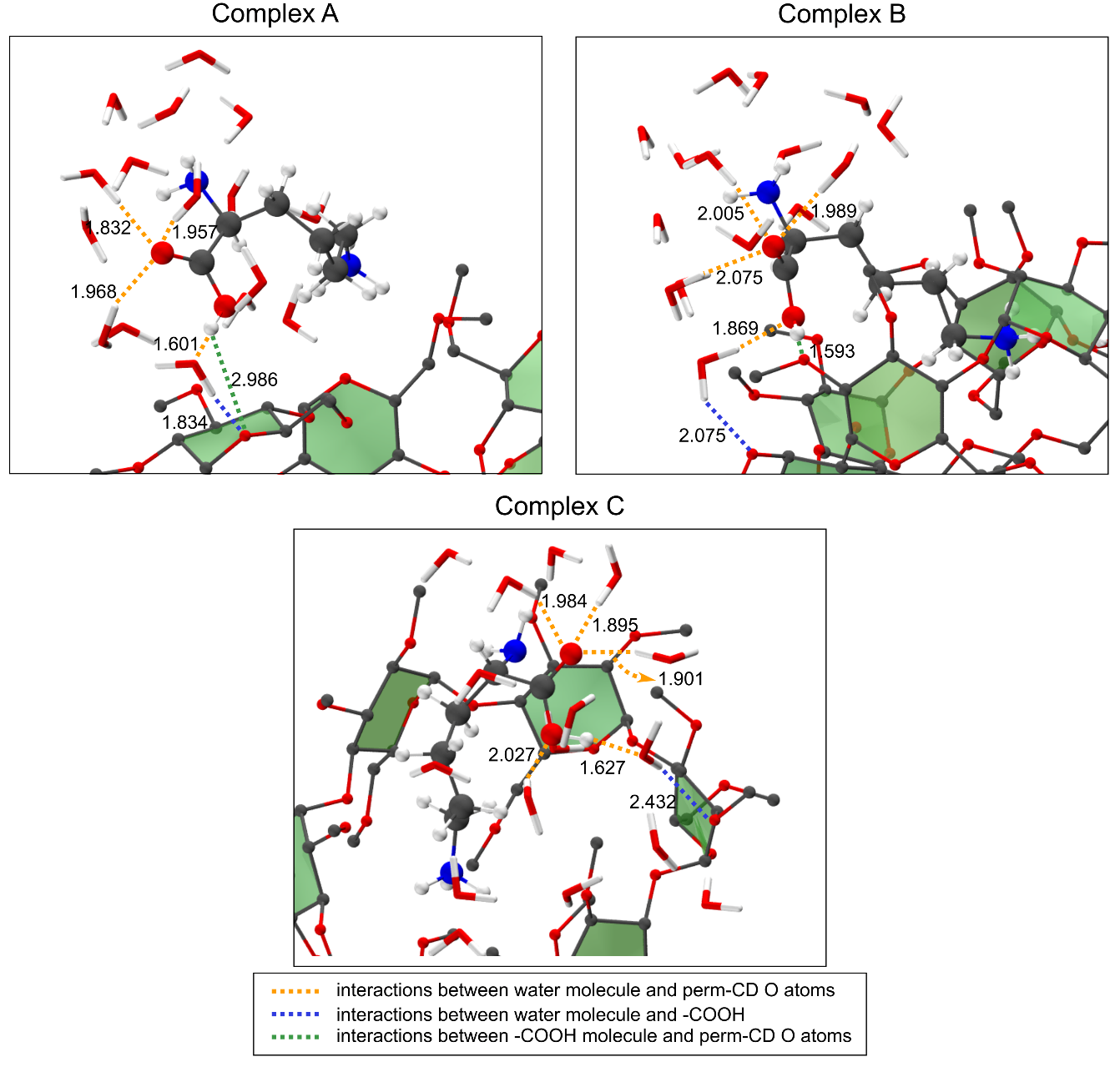
Figure S3. Detailed descriptions of interactions between –COOH, water molecule and perm-CD unit.

Comparison of interactions of -COOH in Complexes A, B and C

- Number of interactions between water molecules and perm-CD O atoms in A, B and C: 1, 1, 1, respectively.
- Number of interactions between water molecules and -COOH in A, B and C: 4, 4, 5, respectively.
- Number of interactions between -COOH and perm-CD O atoms in A, B and C: 1, 1, 0, respectively.

1. In complex A, a water molecule connects the perm-CD and -COOH, the distance between the water molecule and is 1.601Å, and the distance between the water molecule and perm-CD is 1.834Å.
2. In complex B, a water molecule connects the perm-CD and -COOH, the distance between the water molecule and is 1.869Å, and the distance between the water molecule and perm-CD is 2.075Å.
3. In complex C, a water molecule connects the perm-CD and -COOH, the distance between the water molecule and is 1.627Å, and the distance between the water molecule and perm-CD is 2.432Å.

Fig. S4.


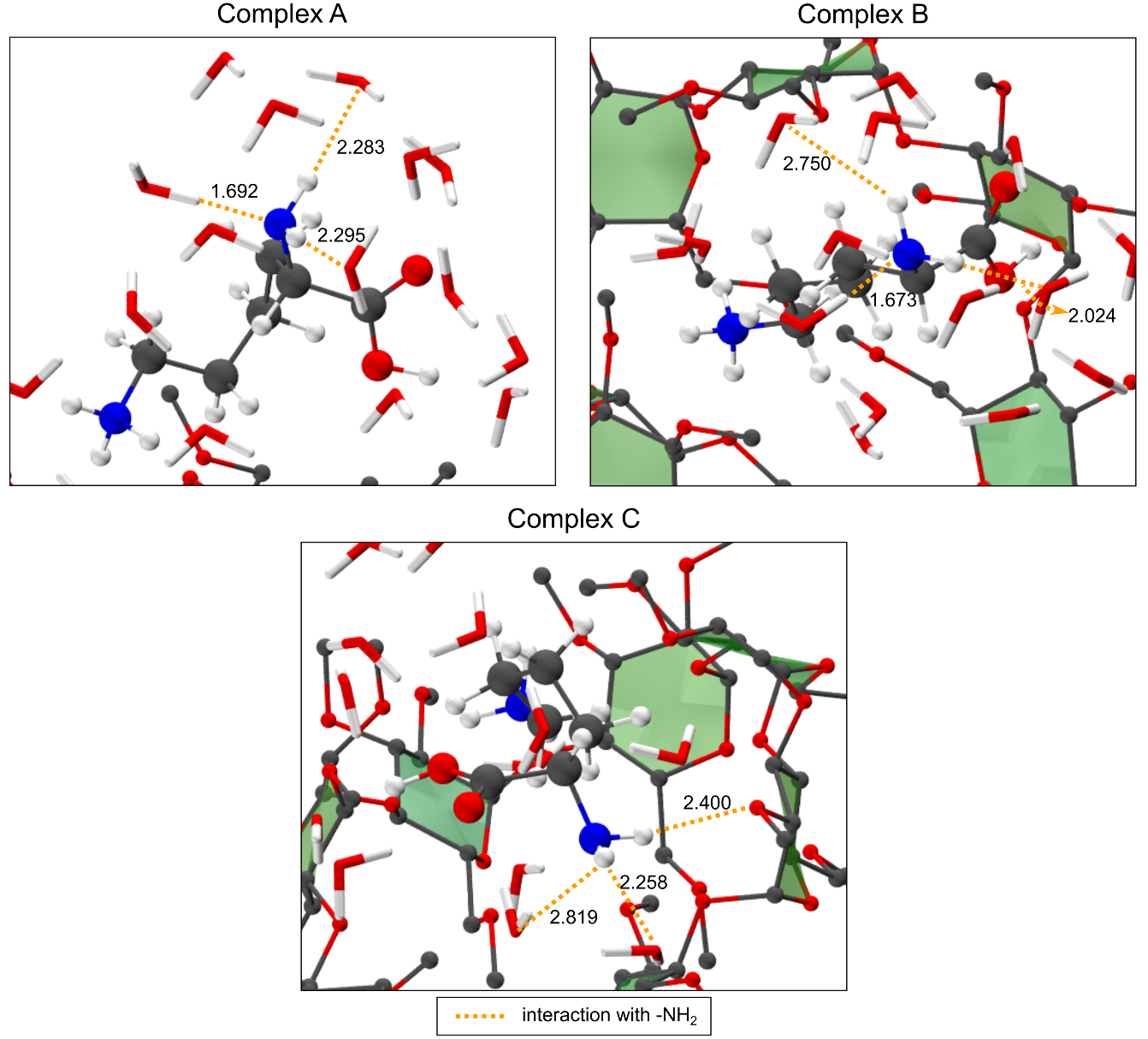


Figure S4. Detailed descriptions of interactions between –NH_2_, water molecule and perm-CD unit.

Comparison of interactions with -NH_2_ in Complexes A, B and C

- Number of water molecules and -NH2 in Complex A, B and C: 3, 3, 2, respectively.

1. In Complex A, distances between water molecules and -NH_2_ are 1.692, 2.295, 2.283 (average = 2.090 Å)
2. In Complex B, distances between water molecules and -NH_2_ are 1.673, 2.024, 2.750 (average = 2.149 Å)
3. In Complex C, distances between water molecules and -NH_2_ are 2.258, 2.819 (average = 2.539Å); Distance between -NH_2_ and perm-CD atom is 2.400Å

Fig. S5.


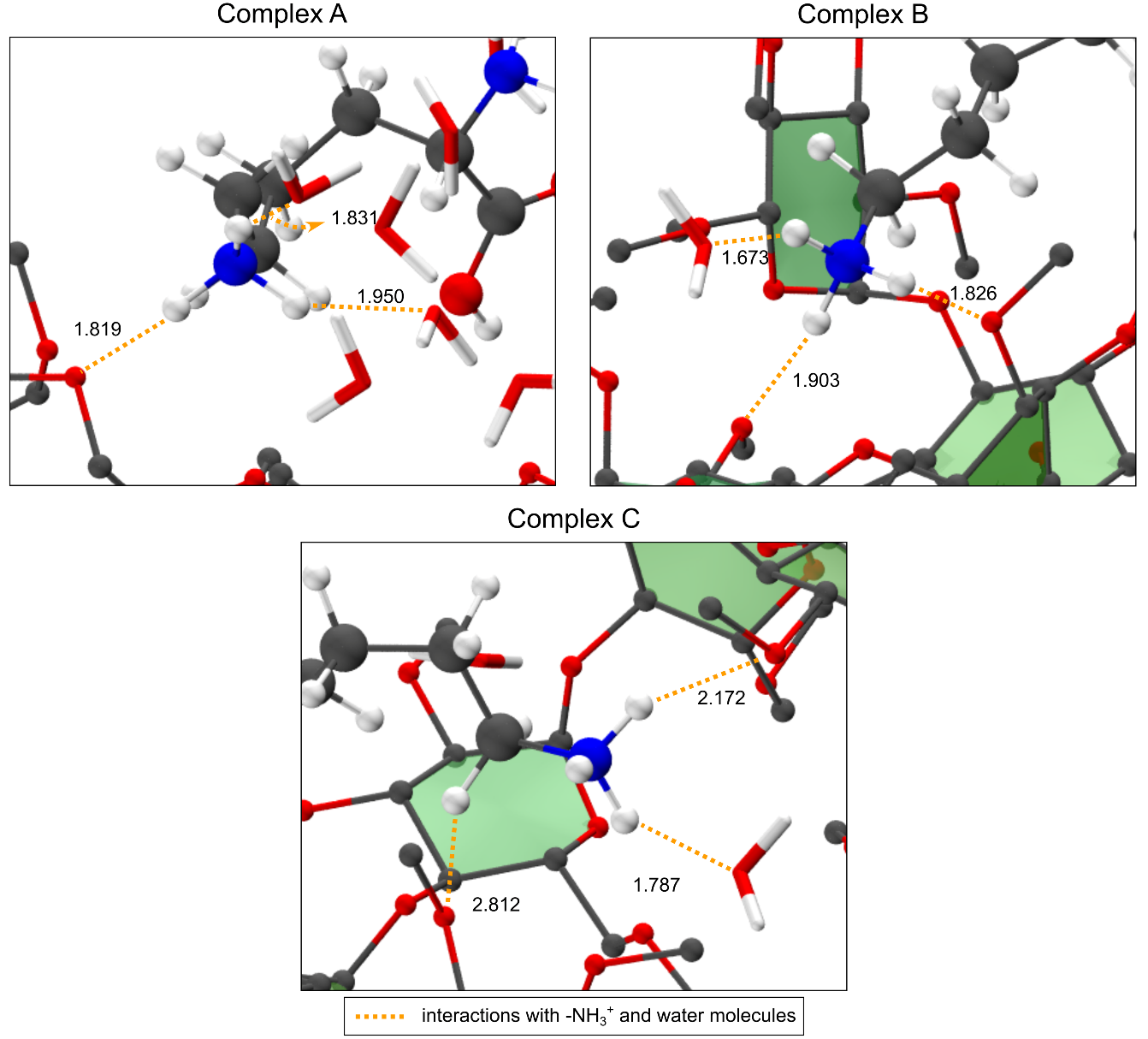


Figure S5. Detailed descriptions of interactions between –NH_3_^+^, water molecule and perm-CD unit.

Comparison of interactions between -NH­_3_^+^ and water molecules in Complexes A, B and C

1. In Complex A, -NH_3_^+^ interacts with 2 water molecules with distances 1.950, 1.831Å also interacts with a perm-CD O atom with distance 1.819Å
2. In Complex B, -NH_3_^+^ interacts with a water molecule and two perm-CD O atoms, distances 1.673, 1.826, 1.903 Å, respectively.
3. In Complex C, -NH_3_^+^ interacts with a water molecule and two perm-CD O atoms, distances 1.787, 2.172, 2.812 Å, respectively.

Table S1.

| Wavenumber (cm^-1^) | Intensity |
| --- | --- |
| 21.95  25.65  48.90  53.55  66.58  75.13  101.31  123.88  141.16  198.96  210.52  230.87  259.21  274.06  301.06  310.99  343.78  347.33  390.64  474.94  504.98  537.83  611.55  682.82  695.78  759.91  823.05  834.06  877.22  900.27  925.47  959.40  984.30  993.56  1046.46  1055.70  1083.22  1112.43  1133.47  1160.99  1185.34  1213.91  1238.82  1258.05  1272.18  1291.77  1309.07  1312.18  1324.57  1348.80  1362.42  1412.64  1422.60  1430.46  1440.77  1522.76  1565.15  1583.29  1589.29  1751.51  2872.03  2880.97  2889.91  2896.78  2908.27  2922.99  2933.88  2949.89  2958.67  2962.78  3052.03  3322.57  3348.26  3393.19  3552.22 | 2.08  0.75  1.50  2.03  0.58  2.71  3.42  12.08  14.12  15.64  10.78  35.53  4.10  32.47  15.69  6.18  2.47  9.34  10.37  7.66  81.60  56.26  94.45  12.96  16.94  8.01  12.81  20.48  33.63  68.85  64.35  2.04  2.80  5.33  1.86  0.48  27.34  194.28  10.77  18.06  8.07  1.72  12.75  5.52  4.21  1.37  0.42  10.60  7.05  5.71  1.87  10.40  14.19  3.88  2.50  135.63  26.44  46.27  241.10  313.45  12.27  16.67  36.85  3.23  25.89  15.96  7.32  619.08  8.59  31.15  547.81  3.42  100.28  6.15  127.43 |

Table S1. Calculated IR frequencies for Complex I (gas phase).

Cartesian coordinates of Complex I (gas phase)

H 0.5947370000 4.2929590000 -2.9859820000

C 2.7591810000 3.0239810000 -3.2786700000

C 3.3179750000 1.8287130000 -4.0510450000

C 4.0975640000 0.8658710000 -3.1522620000

C 4.2811780000 2.7930980000 -1.4808210000

C 5.1785710000 3.6718860000 -0.6173770000

O 2.2657380000 1.1803380000 -4.7162100000

O 4.8700760000 0.0865270000 -4.0390810000

O 3.8361150000 3.5985070000 -2.5696750000

O 6.5064060000 3.6377450000 -1.0846000000

H 2.4246210000 3.7964680000 -3.9798930000

H 4.0326540000 2.2466340000 -4.7744020000

H 3.3687060000 0.2303680000 -2.6231940000

H 3.4183280000 2.5000780000 -0.8729810000

H 4.7765720000 4.6921100000 -0.6522870000

H 5.1270850000 3.3376640000 0.4280660000

C -1.3972390000 4.2832140000 -0.2306570000

C -1.7537110000 3.6815310000 -1.5871670000

C -0.6363350000 2.7787660000 -2.0948320000

C 0.6746140000 3.5698560000 -2.1605450000

C 0.9486590000 4.3038000000 -0.8256310000

C 1.9810740000 5.4133450000 -0.9501250000

O -2.9689440000 2.9733700000 -1.4835450000

O -1.0176230000 2.2996660000 -3.3574180000

O 1.7096520000 2.6306920000 -2.4511700000

O -0.1986620000 4.9826710000 -0.3187010000

O 2.5762810000 5.6260160000 0.3151480000

H -2.1346970000 5.0284520000 0.0558690000

H -1.8634570000 4.5185090000 -2.2907030000

H -0.5080610000 1.9503380000 -1.3835050000

H 1.2853870000 3.5445490000 -0.1111740000

H 2.7518730000 5.1710200000 -1.6867700000

H 1.4488540000 6.3179710000 -1.2799780000

C -4.5883150000 3.4671010000 2.6824210000

C -3.5415300000 3.9228400000 1.6645970000

C -2.1569560000 3.2789890000 1.8864390000

C -2.2623970000 1.7901610000 2.2930070000

C -0.9606590000 1.3384730000 2.9409760000

O -5.7998230000 4.0274650000 2.2595130000

O -3.4585270000 5.3331820000 1.5784400000

O -1.3386490000 3.2444750000 0.7264420000

O -3.3103200000 1.5405630000 3.2170930000

O -0.9723860000 -0.0378920000 3.3412710000

H -4.3372180000 3.8180940000 3.6970690000

H -3.8902100000 3.5958730000 0.6805720000

H -1.6401750000 3.8378880000 2.6827680000

H -2.4395850000 1.2215960000 1.3734010000

H -0.1525830000 1.4750530000 2.2178760000

H -0.7465180000 1.9612940000 3.8208080000

C 7.3288700000 4.5610920000 -0.4174450000

H 6.9689580000 5.5910710000 -0.5573700000

H 7.3760900000 4.3542450000 0.6635110000

H 8.3297340000 4.4708680000 -0.8437210000

C 3.2753870000 6.8423880000 0.3804610000

H 3.7240620000 6.9081100000 1.3742370000

H 4.0743520000 6.8953440000 -0.3761710000

H 2.6016190000 7.6989190000 0.2323250000

C -1.4281490000 -0.2341220000 4.6750980000

H -2.4204820000 0.1955860000 4.8190330000

H -1.4746290000 -1.3145510000 4.8430810000

H -0.7279090000 0.2126510000 5.3951030000

C -6.9306810000 3.6692380000 3.0205540000

H -6.7338800000 3.7430520000 4.1004390000

H -7.7201500000 4.3752690000 2.7566980000

H -7.2762120000 2.6555880000 2.7787640000

C -3.3909180000 6.0567600000 2.7848250000

H -4.3604420000 6.0681370000 3.2980490000

H -2.6233370000 5.6707080000 3.4711810000

H -3.1249580000 7.0793860000 2.5118050000

C -3.9352770000 3.3851570000 -2.4266040000

H -3.5658400000 3.2542690000 -3.4503390000

H -4.8135090000 2.7549910000 -2.2751000000

H -4.2240580000 4.4346780000 -2.2709830000

C -0.7748830000 0.9248070000 -3.5815900000

H -1.1216410000 0.3182680000 -2.7345500000

H -1.3597940000 0.6445040000 -4.4598960000

H 0.2863620000 0.7296120000 -3.7617940000

C 2.5826430000 0.6302370000 -5.9773730000

H 1.6456180000 0.2443580000 -6.3869300000

H 2.9754900000 1.3983290000 -6.6587890000

H 3.3160040000 -0.1779250000 -5.8952250000

C 5.4892460000 -1.0647060000 -3.5008650000

H 4.7865180000 -1.6390520000 -2.8856560000

H 5.8047730000 -1.6667810000 -4.3576340000

H 6.3603080000 -0.8211300000 -2.8837100000

C -6.0516500000 -1.8571140000 -0.4755840000

C -7.0226560000 -0.6901550000 -0.6256160000

C -6.4103450000 0.5846770000 -0.0516920000

C -5.7745780000 0.3705560000 1.3231470000

C -5.0445220000 -0.9709580000 1.4790660000

C -4.7825070000 -1.3248200000 2.9448530000

O -7.3271800000 -0.5432700000 -1.9901640000

O -7.4210760000 1.5662460000 0.0665160000

O -4.8700280000 1.4514380000 1.4847100000

O -5.7871210000 -2.0400130000 0.9021880000

O -5.7998610000 -0.9738760000 3.8479730000

H -6.5022710000 -2.7878890000 -0.8294950000

H -7.9256740000 -0.9440910000 -0.0513860000

H -5.6206880000 0.9156180000 -0.7374980000

H -6.5725070000 0.4377320000 2.0712350000

H -4.0766640000 -0.8888390000 0.9659570000

H -4.5882720000 -2.4067780000 2.9982420000

H -3.8941880000 -0.7800740000 3.2722510000

C -1.6741980000 -3.7593680000 -2.5442160000

C -2.2952530000 -2.7863210000 -3.5390610000

C -3.2389220000 -1.8124460000 -2.8367800000

C -4.2634260000 -2.5641320000 -1.9746980000

C -3.5740160000 -3.6309530000 -1.1008700000

C -4.5319790000 -4.5924450000 -0.3923790000

O -1.2591660000 -2.1219960000 -4.2115120000

O -3.8360590000 -1.0416940000 -3.8415000000

O -4.8754130000 -1.5654890000 -1.1733010000

O -2.6805410000 -4.4525080000 -1.8551820000

O -5.6235470000 -5.0195630000 -1.1648700000

H -1.0951920000 -4.5167230000 -3.0789860000

H -2.8865700000 -3.3917700000 -4.2422350000

H -2.6436530000 -1.1749800000 -2.1655510000

H -5.0112600000 -3.0294980000 -2.6275000000

H -3.0017640000 -3.0841430000 -0.3366750000

H -3.9400060000 -5.4556600000 -0.0462280000

H -4.9618410000 -4.0907970000 0.4766030000

C 2.7177690000 -3.3768690000 0.3916590000

C 2.7543450000 -3.7978310000 -1.0842870000

C 1.5552210000 -3.1904450000 -1.8121710000

C 0.2868240000 -3.7433860000 -1.1726960000

C 0.2998440000 -3.5707880000 0.3531040000

C -0.7085730000 -4.4906610000 1.0239340000

O 3.9768030000 -3.4363850000 -1.6682160000

O 1.5579350000 -3.5254800000 -3.1756450000

O -0.8433320000 -3.0522080000 -1.6595420000

O 1.5489420000 -3.8978290000 0.9725910000

O -1.0166830000 -4.0711600000 2.3501750000

H 3.5536780000 -3.8090340000 0.9428430000

H 2.6569590000 -4.8941180000 -1.0861360000

H 1.5716700000 -2.1008010000 -1.6819100000

H 0.2173620000 -4.8133680000 -1.4180390000

H 0.0475380000 -2.5164170000 0.5470510000

H -0.2616680000 -5.4850330000 1.1044590000

H -1.6185370000 -4.5638490000 0.4234880000

C 5.6922960000 0.7055270000 0.1241730000

C 6.2729470000 -0.6311070000 0.5968640000

C 5.1892430000 -1.7132940000 0.5296500000

C 3.8307220000 -1.2613050000 1.0845820000

C 3.5008490000 0.2161300000 0.8216690000

C 2.4106350000 0.7277550000 1.7465980000

O 7.3376450000 -1.0753230000 -0.2038910000

O 5.5435530000 -2.8601080000 1.2929540000

O 2.7033760000 -1.9686210000 0.5433900000

O 4.6353390000 1.0389990000 1.0131710000

O 2.0111110000 2.0326780000 1.4229000000

H 6.4101400000 1.5275090000 0.1947360000

H 6.6094520000 -0.5066720000 1.6369620000

H 5.0644890000 -1.9868280000 -0.5213530000

H 3.8707000000 -1.4273000000 2.1667290000

H 3.1418570000 0.3012240000 -0.2089310000

H 1.5373100000 0.0729390000 1.6335650000

H 2.7580590000 0.6791700000 2.7861000000

O 5.2493400000 0.4997010000 -1.1654170000

C -5.3083220000 -5.9399900000 -2.1889320000

H -4.7205190000 -6.7812240000 -1.7959020000

H -6.2579030000 -6.3133320000 -2.5769530000

H -4.7404800000 -5.4742470000 -3.0033990000

C -2.1982280000 -3.2954120000 2.4480640000

H -3.0529900000 -3.8400270000 2.0319340000

H -2.3757940000 -3.1236460000 3.5116700000

H -2.1020260000 -2.3258200000 1.9380220000

C 2.4981670000 3.0247720000 2.3096150000

H 2.0451870000 2.9078380000 3.3056920000

H 3.5891160000 2.9696970000 2.3995870000

H 2.2239560000 3.9928670000 1.8885240000

C -6.9552480000 -1.7841400000 3.7651030000

H -7.6102840000 -1.4820700000 4.5842900000

H -7.4803830000 -1.6575840000 2.8109160000

H -6.6996810000 -2.8474360000 3.8775130000

C -1.4721270000 -1.9371220000 -5.5958280000

H -0.5834670000 -1.4294350000 -5.9788020000

H -1.5797550000 -2.9022990000 -6.1118530000

H -2.3585530000 -1.3244690000 -5.7851410000

C -4.2715520000 0.2522600000 -3.4628530000

H -3.5613340000 0.7197060000 -2.7679380000

H -4.3063480000 0.8471510000 -4.3796680000

H -5.2667640000 0.2190300000 -3.0089530000

C -8.6631060000 -0.1687030000 -2.2621500000

H -8.9176760000 0.7861160000 -1.7910740000

H -8.7467330000 -0.0790130000 -3.3468810000

H -9.3683410000 -0.9367520000 -1.9133430000

C -7.1227070000 2.8076210000 -0.5379470000

H -7.0157240000 2.6984110000 -1.6266980000

H -7.9706680000 3.4648270000 -0.3321280000

H -6.2192150000 3.2553760000 -0.1119240000

C 8.5140610000 -0.3032600000 -0.0851800000

H 8.4062040000 0.6834030000 -0.5549970000

H 8.7988210000 -0.1721760000 0.9686370000

H 9.3033850000 -0.8510580000 -0.6025920000

C 6.4640540000 -3.7489270000 0.6768080000

H 6.1981190000 -3.9154970000 -0.3725660000

H 7.4852950000 -3.3634650000 0.7256040000

H 6.3931880000 -4.6932760000 1.2232590000

C 4.4851890000 -4.3862230000 -2.5904500000

H 5.4176050000 -3.9734860000 -2.9800070000

H 4.6973920000 -5.3407410000 -2.0889230000

H 3.7805880000 -4.5535890000 -3.4097660000

C 1.8207480000 -2.4296960000 -4.0386240000

H 1.7407800000 -2.8161490000 -5.0567450000

H 1.0796840000 -1.6364000000 -3.8983900000

H 2.8287580000 -2.0263540000 -3.8823740000

C -4.5861980000 1.9394690000 2.7536730000

H -5.2897630000 1.5499340000 3.4953520000

C 4.9698470000 1.5571560000 -2.0851540000

H 5.9046050000 1.8840780000 -2.5513170000

C 4.8131890000 -1.9038260000 4.8443730000

H 4.3634250000 -2.3751180000 3.9605090000

C 3.6984230000 -1.4905270000 5.8244390000

C 5.5765350000 -0.7292050000 4.2418410000

H 3.5637530000 -2.3444180000 6.4952110000

H 4.0464300000 -0.6682510000 6.4564710000

C 2.3435970000 -1.0992520000 5.2153100000

O 6.7620470000 -0.7040110000 4.0575540000

H 1.6273680000 -0.9886080000 6.0363990000

H 2.4124370000 -0.1227010000 4.7358630000

C 1.7764000000 -2.0902740000 4.1931140000

H 0.7531460000 -1.7981190000 3.9377850000

H 2.3347440000 -2.0337440000 3.2546460000

C 1.8196380000 -3.5233590000 4.7002810000

H 2.8412430000 -3.8469610000 4.9101370000

H 1.2292510000 -3.6494150000 5.6102130000

N 1.2678300000 -4.4563390000 3.6736900000

H 1.3945980000 -5.4313090000 3.9353460000

H 1.6842930000 -4.2957480000 2.7366400000

O 4.7469400000 0.2499600000 3.8494760000

H 5.2063610000 0.8526280000 3.2464150000

N 5.6714920000 -2.9059020000 5.4462450000

H 6.4479070000 -3.0970530000 4.8216650000

H 6.0860050000 -2.5384180000 6.2971160000

H 0.2587860000 -4.2879070000 3.4674920000

Cartesian coordinates of Complex II (gas phase)

H 1.2175410000 -5.7956230000 -0.3848920000

C 2.5637310000 -4.3381220000 1.1697590000

C 2.8052950000 -3.2805450000 2.2458390000

C 3.5600460000 -2.0644400000 1.7061800000

C 4.2845990000 -3.5061670000 -0.2087630000

C 5.3550530000 -4.0627290000 -1.1290720000

O 1.5644190000 -2.9014510000 2.7999910000

O 3.8969900000 -1.3181850000 2.8528190000

O 3.7624280000 -4.6221020000 0.5050500000

O 6.5474450000 -4.2935960000 -0.4146440000

H 2.2461980000 -5.2726460000 1.6323860000

H 3.4425060000 -3.7444860000 3.0107440000

H 2.8769120000 -1.4805000000 1.0659700000

H 3.4994390000 -3.0779310000 -0.8410730000

H 4.9651350000 -5.0024660000 -1.5463090000

H 5.5313880000 -3.3723650000 -1.9674710000

C -2.0621490000 -4.9243290000 -1.1418660000

C -1.6847910000 -5.6732380000 0.1424670000

C -0.4355870000 -5.0791160000 0.7822730000

C 0.6916290000 -4.8437870000 -0.2285430000

C 0.1981490000 -4.2997730000 -1.5742530000

C 1.1793420000 -4.4870560000 -2.7284590000

O -2.6932830000 -5.6607520000 1.1164370000

O 0.0772610000 -5.9494310000 1.7696570000

O 1.5799780000 -3.8664840000 0.2908290000

O -0.9767900000 -4.9655270000 -2.0309980000

O 2.2351680000 -3.5697980000 -2.6230040000

H -2.8761640000 -5.4371010000 -1.6626430000

H -1.4565240000 -6.7078620000 -0.1575480000

H -0.6887250000 -4.1097420000 1.2273080000

H -0.0012850000 -3.2322990000 -1.4306400000

H 1.5553750000 -5.5225510000 -2.7430730000

H 0.6235810000 -4.3242140000 -3.6657370000

C -5.6414890000 -2.1349330000 -2.1307870000

C -4.7290410000 -2.9761460000 -1.2392460000

C -3.2927490000 -2.9742550000 -1.7746420000

C -2.8088750000 -1.5367280000 -1.9980980000

C -1.4424520000 -1.4308120000 -2.6759700000

O -6.9032580000 -2.1233610000 -1.5268430000

O -5.1879090000 -4.2972070000 -1.0257950000

O -2.4378870000 -3.6099470000 -0.8364900000

O -3.7273550000 -0.8701130000 -2.8596270000

O -0.4947860000 -0.9044070000 -1.7695390000

H -5.7072460000 -2.5814740000 -3.1382670000

H -4.7373030000 -2.5211370000 -0.2436640000

H -3.2418140000 -3.4996950000 -2.7385970000

H -2.7599480000 -1.0213490000 -1.0296740000

H -1.1097740000 -2.4087890000 -3.0528640000

H -1.5464340000 -0.7588140000 -3.5406530000

C 7.5358870000 -4.9071490000 -1.2060660000

H 7.1983580000 -5.8847710000 -1.5786410000

H 7.8076870000 -4.2789030000 -2.0689280000

H 8.4151650000 -5.0492780000 -0.5750470000

C 3.1086150000 -3.5984820000 -3.7263920000

H 3.8068470000 -2.7692430000 -3.5959120000

H 3.6548960000 -4.5515200000 -3.7816710000

H 2.5601970000 -3.4599070000 -4.6712310000

C 0.7624730000 -0.7018850000 -2.3891140000

H 1.4387500000 -0.2779470000 -1.6443820000

H 1.2005690000 -1.6417910000 -2.7387360000

H 0.6816010000 0.0086710000 -3.2226900000

C -7.8999770000 -1.4190070000 -2.2323980000

H -7.9038320000 -1.6848030000 -3.3000060000

H -8.8562970000 -1.7027480000 -1.7901280000

H -7.7794240000 -0.3328190000 -2.1314350000

C -5.7222060000 -4.9775790000 -2.1396810000

H -6.6894890000 -4.5585060000 -2.4396550000

H -5.0422330000 -4.9755480000 -3.0047340000

H -5.8714650000 -6.0129870000 -1.8247910000

C -3.7603750000 -6.5596370000 0.9008560000

H -3.3881290000 -7.5825950000 0.7495480000

H -4.3696610000 -6.5267870000 1.8058090000

H -4.3762520000 -6.2545130000 0.0487300000

C -0.3881800000 -5.7182890000 3.0847920000

H -1.4336990000 -6.0209290000 3.2001510000

H 0.2425310000 -6.3195830000 3.7444710000

H -0.2924160000 -4.6606110000 3.3524720000

C 1.5390010000 -2.7985800000 4.2109730000

H 0.5062870000 -2.5664980000 4.4842480000

H 1.8189930000 -3.7510810000 4.6820650000

H 2.2126410000 -2.0114320000 4.5657040000

C 4.4298090000 -0.0286810000 2.6695570000

H 3.8946960000 0.5240700000 1.8784270000

H 4.3030530000 0.4971110000 3.6200060000

H 5.4906910000 -0.0580420000 2.4037840000

C -4.6614000000 3.8879600000 -0.1218680000

C -5.9159920000 3.2248430000 0.4386920000

C -5.8324900000 1.7108750000 0.2432440000

C -5.4827490000 1.3451420000 -1.2038540000

C -4.3912010000 2.2449650000 -1.8122180000

C -4.2988380000 2.1662010000 -3.3393120000

O -6.0235870000 3.5831900000 1.7929230000

O -7.0643290000 1.1492950000 0.6220900000

O -5.0571330000 -0.0152300000 -1.1569050000

O -4.5950940000 3.6212000000 -1.4997140000

O -5.5322000000 2.0719180000 -4.0070430000

H -4.7110340000 4.9735430000 -0.0135630000

H -6.7722420000 3.6162500000 -0.1301040000

H -5.0267590000 1.3352620000 0.8873290000

H -6.3984330000 1.4241640000 -1.8017410000

H -3.4382860000 1.9211950000 -1.3800120000

H -3.7434590000 3.0533150000 -3.6824560000

H -3.7396860000 1.2715650000 -3.6192340000

C 0.3200400000 4.7693630000 1.3538540000

C -0.3878990000 3.9519510000 2.4378520000

C -1.7147350000 3.3270990000 1.9956720000

C -2.5558210000 4.2343340000 1.0846650000

C -1.6403360000 4.8463500000 0.0122710000

C -2.3261520000 5.8087490000 -0.9530620000

O 0.5316260000 2.9594280000 2.8771110000

O -2.3360890000 2.9588660000 3.1997630000

O -3.5269500000 3.3654940000 0.5266640000

O -0.5842840000 5.5641710000 0.6507350000

O -3.2207530000 6.7117530000 -0.3557500000

H 1.0303240000 5.4638560000 1.7991330000

H -0.6139170000 4.6358680000 3.2655060000

H -1.4994820000 2.4345270000 1.3881450000

H -3.0372490000 5.0263130000 1.6702620000

H -1.2082540000 4.0246420000 -0.5757600000

H -1.5379470000 6.3434930000 -1.5061620000

H -2.9196770000 5.2264510000 -1.6620560000

C 4.5460960000 3.7882830000 -1.5308610000

C 4.4933210000 4.8864270000 -0.4573790000

C 3.3280880000 4.6054260000 0.4931820000

C 2.0296940000 4.4167220000 -0.2960740000

C 2.2309450000 3.4190310000 -1.4438330000

C 1.0186930000 3.2641030000 -2.3465230000

O 5.6569610000 4.9530000000 0.3146660000

O 3.1623670000 5.6685560000 1.4021130000

O 1.0023730000 3.8658330000 0.5145080000

O 3.3272210000 3.7992270000 -2.2542980000

O 0.1497380000 2.3082950000 -1.7722960000

H 5.3257590000 3.9724160000 -2.2764100000

H 4.3108380000 5.8469340000 -0.9651750000

H 3.5389420000 3.6712220000 1.0324780000

H 1.7080340000 5.3841750000 -0.7042430000

H 2.4420060000 2.4307990000 -1.0168540000

H 1.3612670000 2.9140550000 -3.3309410000

H 0.4948230000 4.2221660000 -2.4831420000

C 6.1884820000 -1.1309240000 -0.6960100000

C 7.1911110000 -0.0871170000 -0.1955260000

C 6.5430040000 1.2894840000 -0.1280110000

C 5.6772200000 1.6161180000 -1.3467440000

C 4.9100820000 0.4113410000 -1.9218780000

C 4.4170100000 0.6897870000 -3.3259430000

O 7.6657780000 -0.3950030000 1.0927560000

O 7.5015550000 2.3147310000 -0.0199010000

O 4.7362340000 2.5745970000 -0.8845190000

O 5.7224750000 -0.7492020000 -1.9690120000

O 3.4662150000 -0.2843730000 -3.6790250000

H 6.6817790000 -2.0938030000 -0.8202430000

H 8.0236140000 -0.0518810000 -0.9154190000

H 5.8724910000 1.3025540000 0.7391090000

H 6.3253440000 2.0548340000 -2.1178060000

H 4.0422440000 0.2259580000 -1.2767090000

H 3.9657300000 1.6940390000 -3.3600620000

H 5.2717720000 0.6707670000 -4.0210420000

O 5.1171980000 -1.2135820000 0.1983400000

C -2.6207420000 7.7772960000 0.3529660000

H -1.8665510000 8.2851490000 -0.2640630000

H -3.4201750000 8.4804700000 0.5937680000

H -2.1399950000 7.4388510000 1.2777500000

C -1.0082450000 2.1061820000 -2.5519420000

H -0.7471670000 1.8462730000 -3.5876140000

H -1.5486300000 1.2754220000 -2.1014560000

H -1.6357380000 3.0077980000 -2.5612330000

C 2.9838940000 -0.1149620000 -4.9873110000

H 2.4917650000 0.8628210000 -5.1129150000

H 3.7902910000 -0.1954420000 -5.7307570000

H 2.2529190000 -0.9067070000 -5.1659180000

C -6.2917840000 3.2634650000 -4.0171340000

H -7.1184280000 3.1019500000 -4.7116590000

H -6.6904570000 3.5088420000 -3.0251410000

H -5.6871280000 4.1131580000 -4.3630200000

C 0.4245780000 2.5543120000 4.2371160000

H 1.2644940000 1.8805430000 4.4211810000

H 0.5085540000 3.4234660000 4.9000410000

H -0.5219680000 2.0455950000 4.4290130000

C -3.4605450000 2.0961820000 3.1261310000

H -3.3119270000 1.3188040000 2.3675450000

H -3.5461460000 1.6239900000 4.1086760000

H -4.3761700000 2.6431820000 2.8923600000

C -7.3464120000 3.7459300000 2.2689880000

H -7.9216640000 2.8215290000 2.1696200000

H -7.2626800000 4.0216260000 3.3220220000

H -7.8610420000 4.5537740000 1.7293010000

C -6.9750130000 -0.1141800000 1.2598030000

H -6.2461840000 -0.0825400000 2.0823370000

H -7.9660680000 -0.3212780000 1.6710550000

H -6.6883080000 -0.9085970000 0.5674150000

C 8.5430260000 -1.5020230000 1.1460630000

H 8.0283880000 -2.4500820000 0.9401470000

H 9.3708900000 -1.3833410000 0.4326360000

H 8.9479340000 -1.5326430000 2.1589130000

C 7.7810510000 2.7131470000 1.3084630000

H 6.8876080000 3.1506510000 1.7736040000

H 8.1451350000 1.8741930000 1.9091450000

H 8.5575740000 3.4786090000 1.2449400000

C 6.7874760000 5.4609340000 -0.3624510000

H 7.5265010000 5.7051420000 0.4025030000

H 7.2214070000 4.7142610000 -1.0384470000

H 6.5392730000 6.3745420000 -0.9207240000

C 3.7283900000 5.4382300000 2.6783970000

H 3.5502230000 6.3423830000 3.2636900000

H 3.2382240000 4.5855450000 3.1724250000

H 4.8044250000 5.2541430000 2.6069130000

C -5.0371360000 -0.7359730000 -2.3502710000

H -5.5823700000 -0.1988660000 -3.1322140000

C 4.7478890000 -2.4448010000 0.8061140000

H 5.5726410000 -2.8363120000 1.4121640000

C -3.6212540000 -1.9148010000 3.9415850000

H -4.2464460000 -1.3001970000 3.2860970000

C -2.2093310000 -1.3061490000 3.9682640000

C -3.5254520000 -3.3093840000 3.3298050000

H -2.3136060000 -0.2903720000 4.3586200000

H -1.6137470000 -1.8613720000 4.7019280000

C -1.5118890000 -1.2946480000 2.6080160000

O -3.3165960000 -4.3029160000 3.9810900000

H -1.0624340000 -2.2692830000 2.3960880000

H -2.2598870000 -1.1315890000 1.8241020000

C -0.4451810000 -0.2159630000 2.4889860000

H -0.8967630000 0.7470460000 2.7394470000

H 0.3766160000 -0.3923240000 3.1906290000

C 0.0728760000 -0.1773440000 1.0670090000

H 0.6463610000 -1.0748610000 0.8291350000

H -0.7444790000 -0.1243600000 0.3511830000

N 0.9353480000 1.0296610000 0.8096900000

H 0.8186920000 1.7627660000 1.5413990000

H 0.6911190000 1.4756500000 -0.0901880000

O -3.6347900000 -3.2866500000 2.0046190000

H -3.3473240000 -4.1518820000 1.6320210000

N -4.1749750000 -1.8948750000 5.2823830000

H -5.1575680000 -2.1425700000 5.2560910000

H -3.7210090000 -2.6206110000 5.8294710000

H 1.9253330000 0.7880150000 0.7882040000

Cartesian coordinates of Complex III (gas phase)

H 1.2806790000 -5.8230840000 -0.2334730000

C 2.6165470000 -4.3276870000 1.2938530000

C 2.8507820000 -3.2466220000 2.3479900000

C 3.6023960000 -2.0392450000 1.7848790000

C 4.3382590000 -3.5182930000 -0.0969910000

C 5.4136090000 -4.0899700000 -1.0022040000

O 1.6067960000 -2.8607990000 2.8903970000

O 3.9330150000 -1.2675510000 2.9164330000

O 3.8183150000 -4.6209280000 0.6387810000

O 6.6047570000 -4.3009430000 -0.2795970000

H 2.3012800000 -5.2534590000 1.7752450000

H 3.4875160000 -3.6917450000 3.1243900000

H 2.9189010000 -1.4716770000 1.1304850000

H 3.5533240000 -3.1066470000 -0.7404990000

H 5.0285980000 -5.0398640000 -1.4005730000

H 5.5897480000 -3.4168140000 -1.8545220000

C -2.0001100000 -4.9809920000 -1.0182230000

C -1.6236640000 -5.7010420000 0.2827710000

C -0.3786950000 -5.0885810000 0.9134660000

C 0.7506010000 -4.8702390000 -0.0988400000

C 0.2590150000 -4.3567880000 -1.4571920000

C 1.2443650000 -4.5645740000 -2.6043270000

O -2.6350960000 -5.6719430000 1.2533360000

O 0.1345950000 -5.9357630000 1.9205410000

O 1.6335850000 -3.8786550000 0.4022880000

O -0.9119550000 -5.0367040000 -1.9031340000

O 2.2962890000 -3.6411190000 -2.5152660000

H -2.8105690000 -5.5078880000 -1.5303770000

H -1.3904790000 -6.7408800000 0.0053960000

H -0.6369310000 -4.1110110000 1.3371380000

H 0.0549970000 -3.2873100000 -1.3367950000

H 1.6244690000 -5.5986510000 -2.5959200000

H 0.6907650000 -4.4238080000 -3.5464510000

C -5.5873320000 -2.2273430000 -2.0763530000

C -4.6742670000 -3.0458620000 -1.1645610000

C -3.2364070000 -3.0496340000 -1.6957130000

C -2.7574710000 -1.6152510000 -1.9481600000

C -1.3894580000 -1.5183060000 -2.6241640000

O -6.8509230000 -2.2079740000 -1.4764430000

O -5.1286210000 -4.3639260000 -0.9245080000

O -2.3818720000 -3.6619370000 -0.7418370000

O -3.6759700000 -0.9706620000 -2.8262700000

O -0.4465530000 -0.9690720000 -1.7263450000

H -5.6483420000 -2.6953580000 -3.0743350000

H -4.6872660000 -2.5699340000 -0.1788590000

H -3.1805540000 -3.5951530000 -2.6481720000

H -2.7134380000 -1.0793010000 -0.9907310000

H -1.0518540000 -2.5027130000 -2.9792980000

H -1.4934820000 -0.8651730000 -3.5031830000

C 7.5979340000 -4.9271950000 -1.0549840000

H 7.2653260000 -5.9138110000 -1.4077480000

H 7.8698540000 -4.3162800000 -1.9301650000

H 8.4758760000 -5.0524550000 -0.4185530000

C 3.1731240000 -3.6896940000 -3.6152660000

H 3.8677290000 -2.8551240000 -3.5003560000

H 3.7232760000 -4.6415210000 -3.6487660000

H 2.6269880000 -3.5733210000 -4.5644100000

C 0.8117470000 -0.7747490000 -2.3464280000

H 1.4841450000 -0.3324680000 -1.6088860000

H 1.2545390000 -1.7201020000 -2.6747960000

H 0.7305890000 -0.0823210000 -3.1950960000

C -7.8482730000 -1.5226640000 -2.1996380000

H -7.8479130000 -1.8110100000 -3.2613840000

H -8.8047930000 -1.8007620000 -1.7542310000

H -7.7322530000 -0.4341140000 -2.1213530000

C -5.6569450000 -5.0698310000 -2.0252750000

H -6.6249560000 -4.6610310000 -2.3368570000

H -4.9744110000 -5.0834200000 -2.8882060000

H -5.8031070000 -6.0989250000 -1.6889630000

C -3.6980300000 -6.5794040000 1.0537490000

H -3.3213530000 -7.6038530000 0.9252200000

H -4.3101320000 -6.5298160000 1.9560360000

H -4.3125500000 -6.2948220000 0.1935750000

C -0.3356580000 -5.6786800000 3.2291320000

H -1.3803280000 -5.9829450000 3.3478480000

H 0.2954230000 -6.2633770000 3.9032210000

H -0.2448110000 -4.6152030000 3.4746290000

C 1.5767730000 -2.7281860000 4.2988000000

H 0.5423570000 -2.4944610000 4.5641110000

H 1.8590730000 -3.6693860000 4.7907690000

H 2.2462850000 -1.9310470000 4.6387330000

C 4.4613500000 0.0198830000 2.7074470000

H 3.9264470000 0.5536440000 1.9032430000

H 4.3297180000 0.5651680000 3.6461770000

H 5.5231260000 -0.0108990000 2.4454280000

C -4.6366900000 3.8405600000 -0.1926050000

C -5.8903540000 3.1844970000 0.3782380000

C -5.8003750000 1.6670740000 0.2151390000

C -5.4449030000 1.2721800000 -1.2228710000

C -4.3550610000 2.1632370000 -1.8469900000

C -4.2578450000 2.0525350000 -3.3718010000

O -6.0033770000 3.5709990000 1.7242590000

O -7.0311390000 1.1087690000 0.6022250000

O -5.0141340000 -0.0851980000 -1.1458900000

O -4.5652410000 3.5449600000 -1.5642940000

O -5.4888350000 1.9392580000 -4.0409550000

H -4.6908750000 4.9279880000 -0.1074570000

H -6.7464230000 3.5603860000 -0.2011950000

H -4.9951080000 1.3083700000 0.8693650000

H -6.3591040000 1.3349050000 -1.8249450000

H -3.4021840000 1.8524600000 -1.4052690000

H -3.7049050000 2.9343760000 -3.7320460000

H -3.6943820000 1.1543940000 -3.6310940000

C 0.3368620000 4.7727040000 1.2785360000

C -0.3711080000 3.9756270000 2.3775460000

C -1.6941820000 3.3363090000 1.9448600000

C -2.5360790000 4.2207220000 1.0124130000

C -1.6197950000 4.8135210000 -0.0700470000

C -2.3064760000 5.7525500000 -1.0575260000

O 0.5509250000 2.9962780000 2.8403980000

O -2.3176790000 2.9911860000 3.1546770000

O -3.5021510000 3.3364300000 0.4701280000

O -0.5684520000 5.5488670000 0.5561260000

O -3.2063610000 6.6644480000 -0.4820630000

H 1.0431060000 5.4792730000 1.7110610000

H -0.6022580000 4.6760080000 3.1898740000

H -1.4736460000 2.4319350000 1.3569980000

H -3.0223290000 5.0230080000 1.5797100000

H -1.1827670000 3.9812670000 -0.6392910000

H -1.5187170000 6.2785810000 -1.6195420000

H -2.8956140000 5.1530320000 -1.7557440000

C 4.5752800000 3.7475150000 -1.5725090000

C 4.5150310000 4.8679190000 -0.5226820000

C 3.3480750000 4.6024920000 0.4302400000

C 2.0527830000 4.3919880000 -0.3585970000

C 2.2613390000 3.3710280000 -1.4843850000

C 1.0523810000 3.1922510000 -2.3870880000

O 5.6760990000 4.9554230000 0.2511470000

O 3.1755110000 5.6839590000 1.3159680000

O 1.0252060000 3.8543180000 0.4604930000

O 3.3585320000 3.7383210000 -2.2995400000

O 0.1854970000 2.2453110000 -1.7953090000

H 5.3564360000 3.9189120000 -2.3195300000

H 4.3303210000 5.8167330000 -1.0512300000

H 3.5609590000 3.6807530000 0.9898080000

H 1.7285740000 5.3493050000 -0.7880930000

H 2.4749710000 2.3929000000 -1.0359620000

H 1.3992540000 2.8227890000 -3.3628850000

H 0.5252030000 4.1451120000 -2.5454880000

C 6.2343190000 -1.1463800000 -0.6289170000

C 7.2313800000 -0.0882550000 -0.1477500000

C 6.5777180000 1.2868910000 -0.1112790000

C 5.7143030000 1.5842280000 -1.3391580000

C 4.9535790000 0.3645230000 -1.8908650000

C 4.4636090000 0.6112320000 -3.3019350000

O 7.7034040000 -0.3669220000 1.1481320000

O 7.5319430000 2.3179830000 -0.0221310000

O 4.7682180000 2.5485430000 -0.9000620000

O 5.7706220000 -0.7935340000 -1.9110610000

O 3.5176700000 -0.3739370000 -3.6370580000

H 6.7317310000 -2.1097140000 -0.7313020000

H 8.0658790000 -0.0649710000 -0.8658150000

H 5.9045790000 1.3156590000 0.7534240000

H 6.3630050000 2.0090860000 -2.1174590000

H 4.0845520000 0.1894020000 -1.2444360000

H 4.0085250000 1.6127450000 -3.3586210000

H 5.3205060000 0.5808870000 -3.9939990000

O 5.1607060000 -1.2143280000 0.2638780000

C -2.6126190000 7.7471180000 0.2056610000

H -1.8585770000 8.2447790000 -0.4197980000

H -3.4154980000 8.4520630000 0.4292020000

H -2.1333140000 7.4302290000 1.1387940000

C -0.9694260000 2.0222500000 -2.5737900000

H -0.7042310000 1.7414820000 -3.6029900000

H -1.5078770000 1.1990330000 -2.1074110000

H -1.6003740000 2.9209390000 -2.6040080000

C 3.0385940000 -0.2341650000 -4.9500310000

H 2.5430350000 0.7387860000 -5.0977380000

H 3.8475070000 -0.3271700000 -5.6892670000

H 2.3112430000 -1.0323990000 -5.1139460000

C -6.2530210000 3.1273100000 -4.0784760000

H -7.0769570000 2.9478600000 -4.7718140000

H -6.6556000000 3.3920500000 -3.0930600000

H -5.6506520000 3.9718780000 -4.4405270000

C 0.4414610000 2.6196060000 4.2083520000

H 1.2834180000 1.9532110000 4.4090740000

H 0.5200540000 3.5029240000 4.8529600000

H -0.5036880000 2.1113310000 4.4082380000

C -3.4385440000 2.1226990000 3.0960700000

H -3.2846420000 1.3300270000 2.3545560000

H -3.5252310000 1.6710720000 4.0881470000

H -4.3555920000 2.6609970000 2.8481210000

C -7.3282370000 3.7385420000 2.1929390000

H -7.8995870000 2.8099760000 2.1115050000

H -7.2487150000 4.0367950000 3.2401370000

H -7.8444000000 4.5327400000 1.6347780000

C -6.9388020000 -0.1405620000 1.2668080000

H -6.2125530000 -0.0886370000 2.0905940000

H -7.9302640000 -0.3428310000 1.6794830000

H -6.6469460000 -0.9483150000 0.5922330000

C 8.5847940000 -1.4690880000 1.2274120000

H 8.0744700000 -2.4233210000 1.0401310000

H 9.4143110000 -1.3622590000 0.5140310000

H 8.9868010000 -1.4766610000 2.2418510000

C 7.8059280000 2.7455260000 1.2983010000

H 6.9094050000 3.1892410000 1.7514840000

H 8.1714840000 1.9209230000 1.9176670000

H 8.5796480000 3.5125400000 1.2208290000

C 6.8066380000 5.4533790000 -0.4332990000

H 7.5424250000 5.7166450000 0.3284470000

H 7.2454860000 4.6942890000 -1.0920720000

H 6.5565430000 6.3539780000 -1.0115120000

C 3.7386220000 5.4829380000 2.5984780000

H 3.5551920000 6.3985660000 3.1639740000

H 3.2503110000 4.6389690000 3.1090320000

H 4.8155740000 5.3016390000 2.5340250000

C -4.9877750000 -0.8309560000 -2.3236630000

H -5.5327660000 -0.3126820000 -3.1183800000

C 4.7943870000 -2.4338580000 0.8965200000

H 5.6188490000 -2.8091900000 1.5131100000

C -3.5775050000 -2.6114280000 2.9073300000

H -2.9831890000 -3.3680790000 3.4510220000

C -2.5888860000 -1.6721290000 2.1893220000

C -4.3896860000 -1.8982810000 3.9737440000

H -2.4074750000 -2.1141870000 1.2053910000

H -3.0804370000 -0.7092400000 2.0134660000

C -1.2647760000 -1.5111330000 2.9354190000

O -5.5780610000 -1.9956560000 4.1116670000

H -1.4529350000 -1.4712440000 4.0097260000

H -0.6491820000 -2.3924350000 2.7410010000

C -0.4537510000 -0.2707530000 2.5717590000

H -0.9891730000 0.6253150000 2.9001250000

H 0.5012470000 -0.3039820000 3.1083350000

C -0.1830670000 -0.1716620000 1.0851970000

H 0.2631530000 -1.0853710000 0.6914770000

H -1.0930890000 0.0161400000 0.5176930000

N 0.7533250000 0.9672240000 0.7814440000

H 0.5697340000 1.3848800000 -0.1485190000

H 1.7223010000 0.6507960000 0.8029730000

O -3.6389400000 -1.1399860000 4.7981360000

H -4.2439730000 -0.7695500000 5.4520850000

N -4.4930360000 -3.1898040000 1.9513030000

H -4.0366930000 -3.9526380000 1.4626990000

H -5.3340560000 -3.5286620000 2.4003790000

H 0.6822170000 1.7351050000 1.4807920000

Cartesian coordinates of Complex A (solution phase)

H -2.7774410000 5.2459400000 1.6536730000

C -0.7907180000 5.7475590000 0.1712990000

C -0.4433630000 5.8939290000 -1.3103970000

C 0.8001830000 5.0913270000 -1.7003650000

C 1.3847440000 5.0492870000 0.7892550000

C 2.4133270000 5.3293180000 1.8786230000

O -1.5589530000 5.5297480000 -2.0822140000

O 1.2163560000 5.6425350000 -2.9322280000

O 0.3776860000 6.0525890000 0.9006370000

O 3.3824040000 6.2445950000 1.4224970000

H -1.5225310000 6.5083180000 0.4643780000

H -0.2012580000 6.9551810000 -1.4522750000

H 0.4979910000 4.0395330000 -1.8316220000

H 0.9421420000 4.0671590000 1.0002060000

H 1.8756500000 5.7250920000 2.7547360000

H 2.8860460000 4.3859730000 2.1975910000

C -3.3122190000 2.3962550000 3.4213500000

C -4.1279210000 2.8606530000 2.2179390000

C -3.2191700000 3.2345320000 1.0536140000

C -2.2229750000 4.3090320000 1.5028060000

C -1.5112610000 3.8944140000 2.8132820000

C -0.8390160000 5.0570940000 3.5265410000

O -5.0367460000 1.8488170000 1.8450140000

O -4.0355870000 3.6887890000 0.0051260000

O -1.2849230000 4.4732350000 0.4380910000

O -2.4098380000 3.3859750000 3.7956950000

O 0.2108480000 4.5541430000 4.3280530000

H -3.9582960000 2.2625330000 4.2896710000

H -4.6736800000 3.7599810000 2.5330010000

H -2.6540710000 2.3388450000 0.7561560000

H -0.7729670000 3.1288310000 2.5489630000

H -0.4458030000 5.7922960000 2.8271860000

H -1.6033980000 5.5376800000 4.1590980000

C -4.7009560000 -1.5520670000 4.7694080000

C -4.3039010000 -0.1105630000 4.4471740000

C -2.8793030000 0.0126920000 3.8674010000

C -2.5471720000 -1.1332390000 2.8827370000

C -1.0385670000 -1.2428560000 2.7080430000

O -6.0481970000 -1.4997750000 5.1464730000

O -4.5009030000 0.7575930000 5.5472340000

O -2.6659150000 1.1835210000 3.0908190000

O -3.0207140000 -2.3962870000 3.3216420000

O -0.6452880000 -2.2798880000 1.7995480000

H -4.0886480000 -1.9633990000 5.5930860000

H -4.9930280000 0.2425050000 3.6787890000

H -2.1635230000 -0.0103690000 4.7083280000

H -3.0163240000 -0.8786830000 1.9303350000

H -0.6684960000 -0.2935090000 2.3215360000

H -0.5627610000 -1.4273420000 3.6860200000

C 4.2508080000 6.6525240000 2.4506820000

H 3.7019200000 7.1466510000 3.2720300000

H 4.8050270000 5.7985000000 2.8825510000

H 4.9593210000 7.3573630000 2.0217800000

C 0.6556570000 5.4890710000 5.2778920000

H 1.4794740000 5.0270250000 5.8338570000

H 1.0156520000 6.4117820000 4.8071580000

H -0.1456550000 5.7507400000 5.9900920000

C -0.4240550000 -3.5336980000 2.4374770000

H -1.3053810000 -3.8547440000 2.9985760000

H -0.2120570000 -4.2640230000 1.6521490000

H 0.4386700000 -3.4801760000 3.1207560000

C -6.6491700000 -2.7464100000 5.4174240000

H -6.0102850000 -3.3796880000 6.0554510000

H -7.5785620000 -2.5383210000 5.9521630000

H -6.8939780000 -3.2889830000 4.4952040000

C -4.0264790000 0.3330370000 6.8046120000

H -4.6566640000 -0.4667170000 7.2239620000

H -2.9833550000 -0.0141380000 6.7760080000

H -4.0823420000 1.2005640000 7.4638500000

C -6.3736600000 2.2965490000 1.7818240000

H -6.4790400000 3.1119820000 1.0626440000

H -6.9763470000 1.4443590000 1.4612380000

H -6.7318730000 2.6253940000 2.7705120000

C -3.6943110000 3.2070490000 -1.2788340000

H -3.5237840000 2.1209400000 -1.2591800000

H -4.5510830000 3.4048510000 -1.9196210000

H -2.8051540000 3.7094660000 -1.6679080000

C -1.7470270000 6.2614890000 -3.2733530000

H -2.6888640000 5.9119430000 -3.6952090000

H -1.8272170000 7.3379010000 -3.0640770000

H -0.9292730000 6.0963340000 -3.9782800000

C 2.1903180000 4.9177560000 -3.6555770000

H 1.9397520000 3.8510490000 -3.6916260000

H 2.1903990000 5.3358410000 -4.6595590000

H 3.1929770000 5.0121790000 -3.2151650000

C -6.5124340000 -2.8985750000 -1.1776260000

C -7.6268230000 -2.5988430000 -0.1798930000

C -7.0428840000 -2.0289080000 1.1099550000

C -5.8386030000 -2.8238400000 1.6179290000

C -4.9052560000 -3.3325980000 0.5102300000

C -3.9671410000 -4.4379160000 1.0007820000

O -8.5238720000 -1.6965580000 -0.7794730000

O -8.0431670000 -2.0365540000 2.1088190000

O -5.1478180000 -1.9357650000 2.4818820000

O -5.6395770000 -3.8471760000 -0.5963230000

O -4.5220120000 -5.3449220000 1.9181110000

H -6.9112950000 -3.3634270000 -2.0808220000

H -8.1255480000 -3.5544440000 0.0452600000

H -6.7040820000 -1.0081520000 0.9043690000

H -6.2211980000 -3.6792810000 2.1909220000

H -4.2917100000 -2.4876560000 0.1740400000

H -3.5929760000 -4.9786040000 0.1192090000

H -3.1280920000 -3.9687890000 1.5217640000

C -3.2566800000 -0.4865520000 -4.4398790000

C -4.4046980000 0.4614110000 -4.1147890000

C -5.0858770000 0.0684190000 -2.8057030000

C -5.4835240000 -1.4146830000 -2.8160480000

C -4.3133950000 -2.2974770000 -3.2940280000

C -4.6858540000 -3.7565740000 -3.5704980000

O -3.8990720000 1.7688810000 -4.0733930000

O -6.1843560000 0.9242240000 -2.6561900000

O -5.8329590000 -1.7125490000 -1.4738480000

O -3.7251910000 -1.8075040000 -4.5013500000

O -5.9203030000 -3.9425670000 -4.2142530000

H -2.8557990000 -0.2598190000 -5.4283830000

H -5.1350900000 0.3564040000 -4.9255910000

H -4.3673470000 0.2133920000 -1.9801030000

H -6.3529700000 -1.5504130000 -3.4648600000

H -3.5588370000 -2.2776960000 -2.4887440000

H -3.8663260000 -4.2020040000 -4.1594470000

H -4.7606400000 -4.2914850000 -2.6191400000

C 1.8513040000 -0.3445820000 -3.0457400000

C 1.2994270000 0.4813750000 -4.2163250000

C -0.1797380000 0.7788620000 -3.9709240000

C -0.9236250000 -0.5502560000 -3.9109620000

C -0.2650780000 -1.5241390000 -2.9230800000

C -0.7055280000 -2.9570150000 -3.1799390000

O 2.0583570000 1.6461790000 -4.3985320000

O -0.7264430000 1.5571990000 -5.0053720000

O -2.2496190000 -0.3451280000 -3.4724690000

O 1.1634940000 -1.5695290000 -3.0057710000

O -0.4594860000 -3.8017810000 -2.0606250000

H 2.9052210000 -0.5881880000 -3.1943900000

H 1.3957250000 -0.1596630000 -5.1012330000

H -0.2850880000 1.2879680000 -3.0001220000

H -0.9148520000 -0.9929910000 -4.9138670000

H -0.5735230000 -1.2017170000 -1.9128130000

H -0.1099580000 -3.3529330000 -4.0047910000

H -1.7592190000 -2.9893970000 -3.4559840000

C 3.6827340000 3.4239330000 -0.2121460000

C 4.6238080000 2.6268780000 -1.1211260000

C 3.8169830000 1.5906650000 -1.9119100000

C 2.7908090000 0.8279430000 -1.0616050000

C 2.1404780000 1.6683590000 0.0481580000

C 1.5047140000 0.7996920000 1.1192070000

O 5.2805190000 3.4332540000 -2.0665290000

O 4.6555490000 0.5896720000 -2.4766170000

O 1.6735830000 0.3101540000 -1.8034950000

O 3.0895650000 2.4991650000 0.6869230000

O 0.7944180000 1.5577950000 2.0609320000

H 4.2121480000 4.1479120000 0.4193580000

H 5.3614900000 2.1146910000 -0.4765910000

H 3.2884830000 2.1237350000 -2.6989270000

H 3.3336610000 -0.0072160000 -0.5963140000

H 1.3548490000 2.2753380000 -0.4038500000

H 0.7935840000 0.1231820000 0.6328610000

H 2.2798160000 0.1983790000 1.6187560000

O 2.7549570000 4.0179930000 -1.0437980000

C -5.9436710000 -3.5803020000 -5.5787430000

H -5.1101330000 -4.0482560000 -6.1233650000

H -6.8880650000 -3.9435380000 -5.9801870000

H -5.8776110000 -2.4949730000 -5.7172470000

C -1.5848260000 -3.9859790000 -1.2193380000

H -2.4310830000 -4.3803870000 -1.7879360000

H -1.2973780000 -4.7231970000 -0.4618030000

H -1.8840570000 -3.0559100000 -0.7143880000

C 1.4531060000 1.6958850000 3.3092450000

H 1.5139040000 0.7224680000 3.8284960000

H 2.4636180000 2.0971750000 3.1827890000

H 0.8654420000 2.3929190000 3.9108630000

C -5.4435240000 -6.2550020000 1.3516400000

H -5.7072180000 -6.9673130000 2.1386700000

H -6.3486330000 -5.7554520000 0.9930180000

H -4.9922770000 -6.7992740000 0.5096920000

C -4.7294960000 2.7468590000 -4.6627120000

H -4.1971080000 3.6972350000 -4.5654110000

H -4.8913410000 2.5343800000 -5.7253480000

H -5.6933750000 2.8097640000 -4.1493490000

C -6.6131080000 1.1646530000 -1.3267870000

H -5.7551850000 1.2521530000 -0.6423370000

H -7.1462580000 2.1169730000 -1.3396560000

H -7.2825730000 0.3723280000 -0.9743530000

C -9.8831030000 -1.9096170000 -0.4545270000

H -10.0551410000 -1.8384970000 0.6253790000

H -10.4503880000 -1.1369840000 -0.9714930000

H -10.2224470000 -2.8967110000 -0.8024560000

C -8.2519730000 -0.7989980000 2.7564200000

H -8.6211850000 -0.0458010000 2.0505610000

H -9.0163210000 -0.9742640000 3.5192470000

H -7.3405270000 -0.4458330000 3.2501700000

C 6.2432910000 4.3061220000 -1.5118410000

H 5.7789210000 5.1246120000 -0.9408530000

H 6.9403610000 3.7645260000 -0.8469170000

H 6.8016430000 4.7303470000 -2.3409820000

C 5.3408720000 0.9576760000 -3.6633990000

H 4.6715360000 1.4840900000 -4.3436260000

H 6.2011640000 1.5980100000 -3.4408910000

H 5.6780100000 0.0255860000 -4.1207890000

C 2.2549780000 2.0086320000 -5.7541160000

H 2.8392170000 2.9325550000 -5.7420330000

H 2.8240960000 1.2320280000 -6.2808370000

H 1.3016740000 2.1731340000 -6.2524660000

C -1.0564910000 2.8816720000 -4.6159550000

H -1.5117560000 3.3508410000 -5.4840730000

H -1.7763350000 2.8773270000 -3.7881220000

H -0.1632300000 3.4500360000 -4.3186390000

C -4.4160440000 -2.4341340000 3.5531650000

H -4.6436030000 -3.4915480000 3.7356420000

C 1.9275850000 5.1143780000 -0.6488430000

H 2.4924440000 6.0452080000 -0.7496950000

C 5.6622010000 -2.6074090000 1.1541520000

H 5.1787630000 -2.5896490000 0.1725900000

C 4.7304120000 -3.2665260000 2.1953040000

C 5.9321270000 -1.1674410000 1.5376990000

H 4.7915140000 -4.3429900000 2.0047550000

H 5.1607170000 -3.1055220000 3.1912390000

C 3.2625900000 -2.8241950000 2.1942080000

O 6.9914050000 -0.7729240000 2.0291910000

H 2.7111420000 -3.5245000000 2.8326030000

H 3.1647910000 -1.8405260000 2.6626650000

C 2.6097560000 -2.7727160000 0.8127940000

H 1.5550620000 -2.4989300000 0.9182680000

H 3.0776600000 -1.9821420000 0.2153360000

C 2.7195390000 -4.0838150000 0.0493270000

H 3.7502440000 -4.4395630000 -0.0138360000

H 2.1132940000 -4.8730990000 0.4986840000

N 2.2667210000 -3.8919280000 -1.3502200000

H 2.5238630000 -4.6943140000 -1.9537170000

H 2.7658550000 -3.0815320000 -1.7484860000

O 4.9096310000 -0.3804300000 1.3257830000

H 5.0420190000 0.5181430000 1.7716880000

N 6.8716790000 -3.4022060000 1.0441590000

H 7.5330210000 -2.9650000000 0.4053920000

H 7.3195470000 -3.4729550000 1.9562270000

H 1.2476490000 -3.7310650000 -1.4351890000

O 7.1341740000 0.3638190000 -1.0153470000

H 6.2318200000 0.2634160000 -1.3760610000

H 7.6127910000 0.8417860000 -1.7083610000

O 5.1010770000 1.9306970000 2.5237980000

H 4.3657670000 2.2827550000 1.9861940000

H 5.9352320000 2.1897300000 2.0600790000

O 7.5183490000 1.9235600000 1.2864750000

H 7.5780940000 1.0903920000 1.7883350000

H 7.3205800000 1.5770540000 0.3915930000

O 9.0291130000 -1.6959330000 -0.7860520000

H 8.2858850000 -1.0581410000 -0.7789940000

H 9.3024890000 -1.7808640000 0.1475330000

O 6.2508730000 -5.9318730000 0.3359320000

H 7.0441640000 -6.3016030000 -0.0805660000

H 6.5294820000 -4.9942870000 0.5840820000

O 3.4837050000 -5.4729500000 -3.3045650000

H 4.3859150000 -5.3032320000 -2.9412190000

H 3.4927210000 -6.3846250000 -3.6247050000

O 5.8102520000 -4.6602250000 -2.1027460000

H 5.4537000000 -3.7555130000 -1.9644340000

H 5.7399860000 -5.1123840000 -1.2341260000

O 6.8007420000 -1.5537590000 4.7596480000

H 7.0602430000 -1.0630640000 3.9635940000

H 7.1079240000 -2.4603910000 4.5440730000

O 4.3135840000 -0.1790250000 4.4629060000

H 4.6376860000 0.5484300000 3.9068110000

H 5.0920040000 -0.7601880000 4.5670180000

O 8.6221640000 -4.2731380000 -1.6537820000

H 7.6617610000 -4.4037720000 -1.7861410000

H 8.7100920000 -3.3163550000 -1.4438430000

O 4.4674160000 -2.2617160000 -2.2317630000

H 4.1409840000 -2.4279650000 -3.1491270000

H 4.6240840000 -1.3008320000 -2.1797330000

O 8.2484040000 -3.7913000000 4.0173480000

H 8.1299070000 -4.6591760000 3.5638780000

H 8.8442140000 -3.2917170000 3.4278510000

O 9.4720050000 -2.0836250000 2.0348130000

H 8.6294780000 -1.5885550000 2.1156980000

H 10.1542850000 -1.4919870000 2.3823930000

O 3.1470230000 -3.0105590000 -4.5296820000

H 2.3040030000 -2.6967500000 -4.1557580000

H 3.1991330000 -3.9496140000 -4.2481760000

O 8.1455680000 -6.1360750000 2.5792100000

H 7.3080190000 -6.1942860000 2.0883140000

H 8.7442520000 -5.6987600000 1.9251540000

O 9.7471880000 -4.7383370000 0.8512660000

H 9.6865530000 -3.8473990000 1.2407140000

H 9.2791630000 -4.6576280000 -0.0110460000

Cartesian coordinates of Complex B (solution phase)

H -1.1461120000 -5.4479290000 2.1292790000

C -2.1835090000 -4.7253910000 -0.0513690000

C -2.2709260000 -3.9963570000 -1.3913830000

C -3.3805030000 -2.9435840000 -1.4087150000

C -4.3815990000 -4.1307020000 0.5557480000

C -5.5591880000 -4.7637380000 1.2734920000

O -1.0173870000 -3.4156550000 -1.6777320000

O -3.4730780000 -2.5475460000 -2.7577160000

O -3.4517580000 -5.1838320000 0.3237740000

O -6.3921690000 -5.4348220000 0.3562720000

H -1.5589840000 -5.6131260000 -0.1517960000

H -2.5273890000 -4.7502970000 -2.1477960000

H -3.0651170000 -2.0879930000 -0.7874060000

H -3.9386660000 -3.3967390000 1.2374990000

H -5.1470930000 -5.4684720000 2.0102120000

H -6.1249060000 -3.9972590000 1.8240220000

C 1.4841110000 -3.6105040000 3.4688020000

C 1.6899400000 -4.6645190000 2.3736040000

C 0.6193650000 -4.5567250000 1.2942080000

C -0.7943740000 -4.4391010000 1.8732220000

C -0.8720630000 -3.5361760000 3.1097900000

C -2.1135670000 -3.7517340000 3.9713050000

O 2.9334230000 -4.5723450000 1.7324420000

O 0.6285470000 -5.7044140000 0.4707310000

O -1.6435080000 -3.8505020000 0.9003110000

O 0.2020000000 -3.7664470000 4.0184640000

O -3.2382960000 -3.1848610000 3.3541420000

H 2.1731400000 -3.7795430000 4.3015260000

H 1.5846080000 -5.6451770000 2.8632760000

H 0.8067500000 -3.6589560000 0.6936140000

H -0.8497630000 -2.5021180000 2.7490590000

H -2.2647650000 -4.8270980000 4.1573070000

H -1.9288130000 -3.2708520000 4.9452140000

C 3.9437050000 0.1634040000 4.5994600000

C 3.5460310000 -1.0442660000 3.7518520000

C 2.0428310000 -1.3203010000 3.8692660000

C 1.2420760000 -0.0471670000 3.5718350000

C -0.2620720000 -0.1790640000 3.8126400000

O 5.3042710000 0.3927810000 4.3688310000

O 4.2976600000 -2.2120400000 4.0214170000

O 1.6732940000 -2.3274350000 2.9396730000

O 1.6890120000 0.9913490000 4.4389840000

O -0.9547360000 -0.1065130000 2.5831110000

H 3.7706200000 -0.0479330000 5.6690930000

H 3.7823160000 -0.8030330000 2.7105740000

H 1.7908060000 -1.6454600000 4.8884080000

H 1.4048720000 0.2411710000 2.5248320000

H -0.4998180000 -1.1191050000 4.3312540000

H -0.5726020000 0.6493620000 4.4663610000

C -7.4414630000 -6.1247060000 0.9909800000

H -7.0563160000 -6.8876860000 1.6823600000

H -8.0913910000 -5.4375290000 1.5553210000

H -8.0279730000 -6.6129850000 0.2105280000

C -4.3946380000 -3.2317440000 4.1553140000

H -5.1625450000 -2.6581150000 3.6323840000

H -4.7328020000 -4.2664900000 4.3125000000

H -4.2143650000 -2.7742910000 5.1406680000

C -2.3579130000 -0.1293790000 2.7732680000

H -2.8304320000 -0.0507520000 1.7923280000

H -2.6921680000 -1.0666010000 3.2287620000

H -2.6874880000 0.7233960000 3.3819010000

C 5.8661390000 1.4577720000 5.1019550000

H 5.5811710000 1.4123720000 6.1636440000

H 6.9495280000 1.3581650000 5.0182600000

H 5.5744120000 2.4329230000 4.6914570000

C 4.5708570000 -2.4998180000 5.3748090000

H 5.2904700000 -1.7900020000 5.7985570000

H 3.6639580000 -2.5121300000 5.9977620000

H 5.0119040000 -3.4991310000 5.3935730000

C 4.0298950000 -5.0977500000 2.4499760000

H 3.8361700000 -6.1340460000 2.7605650000

H 4.8771460000 -5.0788550000 1.7621840000

H 4.2697720000 -4.4828160000 3.3233830000

C 1.4344670000 -5.6149080000 -0.6877700000

H 2.5000270000 -5.6460460000 -0.4395320000

H 1.1773000000 -6.4767430000 -1.3087610000

H 1.2244620000 -4.6921070000 -1.2389900000

C -0.5639540000 -3.5823220000 -3.0076640000

H 0.4356630000 -3.1418230000 -3.0496710000

H -0.4873990000 -4.6469660000 -3.2689170000

H -1.2293230000 -3.0849610000 -3.7212080000

C -4.2776020000 -1.4349330000 -3.0665610000

H -4.1413140000 -0.6191710000 -2.3359730000

H -3.9595410000 -1.0868040000 -4.0532200000

H -5.3404470000 -1.6928060000 -3.0921010000

C 2.4991590000 5.2101180000 0.8960090000

C 3.9710030000 4.8083230000 0.8807690000

C 4.1268720000 3.3929870000 1.4373490000

C 3.4124640000 3.2322550000 2.7841750000

C 2.0303800000 3.9100930000 2.8247140000

C 1.4731230000 4.1040740000 4.2384290000

O 4.4326310000 4.9157550000 -0.4418490000

O 5.4998560000 3.1159850000 1.5581260000

O 3.2983560000 1.8249400000 2.9851840000

O 2.0503780000 5.2047210000 2.2273940000

O 2.4224900000 4.4697890000 5.2084250000

H 2.3677310000 6.2283740000 0.5237690000

H 4.5073070000 5.5141410000 1.5319870000

H 3.6583400000 2.7010460000 0.7254300000

H 4.0556280000 3.6641880000 3.5600400000

H 1.3476470000 3.2687860000 2.2570200000

H 0.6753690000 4.8610790000 4.1776240000

H 1.0399790000 3.1641940000 4.5854050000

C -1.8224350000 4.4403130000 -2.0195700000

C -0.6605450000 3.6475010000 -2.6244390000

C 0.5510490000 3.4933480000 -1.6999370000

C 0.8634380000 4.7482220000 -0.8704090000

C -0.4471240000 5.2897200000 -0.2771920000

C -0.3068390000 6.5660220000 0.5473060000

O -1.1794740000 2.3817180000 -3.0144160000

O 1.5832110000 3.0790000000 -2.5569590000

O 1.7558480000 4.2914590000 0.1317460000

O -1.3631460000 5.5658380000 -1.3368210000

O 0.5369970000 7.5418060000 -0.0079370000

H -2.4748220000 4.8230390000 -2.8025180000

H -0.3205550000 4.1949490000 -3.5124410000

H 0.3319860000 2.7093440000 -0.9586620000

H 1.3420210000 5.5118740000 -1.4946840000

H -0.8749120000 4.5115140000 0.3701660000

H -1.3186700000 6.9686210000 0.7123840000

H 0.1312530000 6.3089660000 1.5147840000

C -6.4702240000 2.9468650000 -0.1998660000

C -6.2934130000 3.7927130000 -1.4704460000

C -4.8547090000 3.6535560000 -1.9715150000

C -3.8653420000 3.9705780000 -0.8467870000

C -4.2231600000 3.1945710000 0.4271900000

C -3.3564610000 3.5424460000 1.6256060000

O -7.1394380000 3.3992780000 -2.5115920000

O -4.6187750000 4.5277970000 -3.0503580000

O -2.5464810000 3.5666860000 -1.1835180000

O -5.5726080000 3.4188690000 0.7903930000

O -2.1807760000 2.7592720000 1.5727010000

H -7.4670360000 3.0573100000 0.2380730000

H -6.4736700000 4.8461310000 -1.2029580000

H -4.6958550000 2.6114380000 -2.2826280000

H -3.8861060000 5.0483230000 -0.6368680000

H -4.0897830000 2.1228840000 0.2344390000

H -3.9197450000 3.3096960000 2.5407630000

H -3.1012580000 4.6128070000 1.6393060000

C -6.7665920000 -2.2970310000 -0.1175890000

C -7.7407290000 -1.6722350000 -1.1205820000

C -7.3869390000 -0.2125880000 -1.3733760000

C -7.0363910000 0.5591900000 -0.0993830000

C -6.2730300000 -0.2657820000 0.9529830000

C -6.3178500000 0.3972570000 2.3132740000

O -7.7100150000 -2.3360180000 -2.3608290000

O -8.4416910000 0.4815950000 -1.9956640000

O -6.2034840000 1.6256070000 -0.5315760000

O -6.8148860000 -1.5686870000 1.0868140000

O -5.3583860000 -0.2037220000 3.1474920000

H -7.0756840000 -3.3138080000 0.1201600000

H -8.7492850000 -1.7214150000 -0.6813270000

H -6.4911040000 -0.1889100000 -2.0047040000

H -7.9690530000 0.9499570000 0.3301650000

H -5.2262150000 -0.3348170000 0.6317830000

H -6.1063030000 1.4723760000 2.2005620000

H -7.3285490000 0.2861480000 2.7378750000

O -5.4716960000 -2.2628490000 -0.6433400000

C -0.0015680000 8.2538650000 -1.1036520000

H -0.9969870000 8.6523250000 -0.8632050000

H 0.6793400000 9.0839710000 -1.3006760000

H -0.0864420000 7.6293070000 -2.0000970000

C -1.3159480000 3.0245930000 2.6549680000

H -1.8375960000 2.9084400000 3.6155550000

H -0.5088770000 2.2962770000 2.5974010000

H -0.9130120000 4.0446380000 2.5926410000

C -5.3613370000 0.3350680000 4.4446600000

H -5.1359100000 1.4135420000 4.4345860000

H -6.3305950000 0.1879670000 4.9430230000

H -4.5857310000 -0.1852260000 5.0110660000

C 2.8910620000 5.7989720000 5.1056300000

H 3.4678020000 5.9985400000 6.0108020000

H 3.5292310000 5.9462170000 4.2257090000

H 2.0540380000 6.5080840000 5.0441840000

C -0.5673860000 1.7663660000 -4.1422310000

H -1.1560910000 0.8718740000 -4.3579470000

H -0.6035940000 2.4383720000 -5.0077190000

H 0.4710450000 1.4996700000 -3.9370560000

C 2.7710590000 2.5758450000 -1.9655740000

H 2.5431500000 1.9473020000 -1.0967060000

H 3.2560130000 1.9638130000 -2.7310470000

H 3.4389400000 3.3801010000 -1.6504110000

C 5.7795450000 5.3286740000 -0.5773080000

H 6.4618980000 4.6265710000 -0.0907480000

H 5.9838480000 5.3650690000 -1.6491510000

H 5.9269790000 6.3319400000 -0.1525200000

C 5.8670380000 1.7760200000 1.2740980000

H 5.4472650000 1.4550700000 0.3100250000

H 6.9578400000 1.7640040000 1.2082800000

H 5.5355350000 1.0821300000 2.0495470000

C -8.2904410000 -3.6247830000 -2.3513770000

H -7.6936590000 -4.3448690000 -1.7757040000

H -9.3089280000 -3.5951190000 -1.9387940000

H -8.3370070000 -3.9552440000 -3.3903090000

C -8.3551140000 0.5292860000 -3.4069170000

H -7.4635950000 1.0902130000 -3.7176590000

H -8.3373990000 -0.4751880000 -3.8404340000

H -9.2450450000 1.0591140000 -3.7537050000

C -8.5030580000 3.7096420000 -2.3147070000

H -8.9922240000 3.5979230000 -3.2838220000

H -8.9752630000 3.0187250000 -1.6058750000

H -8.6293020000 4.7458310000 -1.9707190000

C -4.6917620000 3.9154060000 -4.3239290000

H -4.5164440000 4.7047790000 -5.0573830000

H -3.9131000000 3.1447210000 -4.4294150000

H -5.6765830000 3.4700850000 -4.4932450000

C 3.0400080000 1.3674140000 4.2766650000

H 3.1914450000 2.1693970000 5.0054220000

C -4.6949110000 -3.4450860000 -0.7869600000

H -5.1901200000 -4.1498010000 -1.4644230000

C 4.2841520000 -1.7308420000 -1.8293010000

H 4.7358230000 -0.9241250000 -1.2424270000

C 2.8907380000 -1.2706320000 -2.2952610000

C 4.1544140000 -2.9162890000 -0.8951500000

H 3.0493670000 -0.3305740000 -2.8325780000

H 2.5036230000 -2.0005550000 -3.0158810000

C 1.9029820000 -1.0530670000 -1.1564810000

O 4.3523710000 -4.0839070000 -1.2169560000

H 1.5881740000 -2.0139180000 -0.7339900000

H 2.4144180000 -0.5132020000 -0.3488730000

C 0.6636390000 -0.2618540000 -1.5432650000

H 0.9461410000 0.6261680000 -2.1152470000

H -0.0142340000 -0.8564240000 -2.1671790000

C -0.0252120000 0.1679960000 -0.2634410000

H -0.3116010000 -0.6905250000 0.3443410000

H 0.6455560000 0.7935170000 0.3314260000

N -1.2466280000 0.9587220000 -0.5346960000

H -1.1895440000 1.4739510000 -1.4295900000

H -1.4639900000 1.6390790000 0.2114520000

O 3.7823000000 -2.5635440000 0.3205060000

H 3.4604160000 -3.3470080000 0.8615310000

N 5.1288860000 -1.9909720000 -2.9802740000

H 6.0471120000 -2.3200500000 -2.6750450000

H 4.7213170000 -2.7179190000 -3.5657140000

H -2.0962840000 0.3795970000 -0.6171710000

O -3.7581600000 0.1955880000 -0.5714640000

H -4.1515460000 -0.6891510000 -0.5439820000

H -4.4721440000 0.7680230000 -0.9033710000

O 6.1223400000 -1.9302540000 1.7398690000

H 5.8628370000 -1.9851580000 2.6785550000

H 5.2624760000 -1.9535590000 1.2847950000

O 6.8675950000 -4.3223670000 0.3703110000

H 6.0457950000 -4.5303600000 -0.1036990000

H 6.6279210000 -3.5415190000 0.9159870000

O 7.7824070000 -2.8236280000 -1.7622510000

H 7.5601730000 -3.4422970000 -1.0294180000

H 7.6344750000 -1.9339610000 -1.3602950000

O 2.6979280000 -4.7851520000 -3.5581560000

H 2.6885910000 -4.0336720000 -4.1893940000

H 3.2727140000 -4.4845320000 -2.8384670000

O 2.8664480000 -2.9252790000 -5.5860270000

H 2.8941500000 -1.9556320000 -5.4695060000

H 3.8097000000 -3.2073740000 -5.6619360000

O 5.3483220000 0.2636930000 -4.4196320000

H 4.4783970000 0.3407950000 -4.8666490000

H 5.2658500000 -0.5953860000 -3.8935920000

O 2.8243380000 -0.0795650000 -5.4826400000

H 2.1219960000 0.2013620000 -4.8791770000

H 2.5487000000 0.2481630000 -6.3503720000

O 6.8097080000 -1.5546600000 -6.0856970000

H 6.2540670000 -0.8845200000 -5.6416650000

H 7.5325170000 -1.7081390000 -5.4334480000

O 8.7960610000 -1.8024780000 -4.1828360000

H 8.6471700000 -0.8583950000 -3.9625870000

H 8.4505670000 -2.2826580000 -3.4066430000

O 8.0238690000 0.8611990000 -3.8437220000

H 8.5295180000 1.4567540000 -4.4140130000

H 7.1808840000 0.7193420000 -4.3199390000

O 6.0267820000 1.5831420000 -2.0581850000

H 6.9199810000 1.5924000000 -2.4594930000

H 5.5210440000 1.1324970000 -2.7685390000

O 5.3740850000 3.8759710000 -3.6865550000

H 5.5681500000 3.2051680000 -3.0045020000

H 4.6245140000 4.3591200000 -3.3156680000

O 7.3444440000 -0.4609830000 -0.4712060000

H 6.7506000000 0.1559050000 -0.9424780000

H 6.8498400000 -0.7697130000 0.3096550000

O 5.4181690000 -3.8860060000 -5.7209830000

H 5.7099410000 -4.2492860000 -4.8576760000

H 5.9405880000 -3.0565010000 -5.8333950000

O 6.3443400000 -4.9018450000 -3.2517280000

H 5.6013490000 -4.7215050000 -2.6499290000

H 6.9958560000 -4.2289680000 -2.9877620000

Cartesian coordinates of Complex C (solution phase)

H 1.6895030000 -5.9110220000 -0.6007690000

C 2.7816210000 -4.5392290000 1.2100150000

C 2.8742940000 -3.4752770000 2.3028090000

C 3.8511220000 -2.3549720000 1.9415660000

C 4.8479320000 -3.9205660000 0.2605580000

C 6.0398300000 -4.6063630000 -0.3810610000

O 1.5837810000 -2.9616210000 2.5504050000

O 3.9886310000 -1.6075740000 3.1280400000

O 4.0662730000 -4.9578090000 0.8442070000

O 7.0066500000 -4.9268400000 0.5926210000

H 2.2740220000 -5.4236680000 1.5951070000

H 3.2686780000 -3.9729640000 3.1989840000

H 3.3956660000 -1.7294600000 1.1549100000

H 4.2762640000 -3.4399790000 -0.5406310000

H 5.6639140000 -5.5175640000 -0.8685310000

H 6.4726090000 -3.9635160000 -1.1621240000

C -1.2237540000 -4.7553470000 -2.1060490000

C -1.2289460000 -5.4948740000 -0.7622380000

C -0.1071160000 -5.0024390000 0.1442950000

C 1.2400700000 -4.9087330000 -0.5796210000

C 1.1263920000 -4.3639280000 -2.0081420000

C 2.3227440000 -4.6813720000 -2.9014360000

O -2.4278690000 -5.3545930000 -0.0489630000

O 0.0749150000 -5.8853300000 1.2318330000

O 2.0787650000 -4.0044730000 0.1224660000

O 0.0271650000 -4.9288200000 -2.7187180000

O 3.4126850000 -3.8662500000 -2.5625400000

H -1.9435120000 -5.2045950000 -2.7966170000

H -1.0429330000 -6.5557680000 -0.9914030000

H -0.3571050000 -3.9994110000 0.5093540000

H 1.0075330000 -3.2781910000 -1.9247310000

H 2.5861020000 -5.7479840000 -2.8187050000

H 2.0165900000 -4.4966150000 -3.9435580000

C -4.1811020000 -1.6702400000 -3.9244450000

C -3.5872860000 -2.5654260000 -2.8377210000

C -2.0736570000 -2.7181470000 -3.0253020000

C -1.4094970000 -1.3414090000 -3.1438910000

C 0.0797020000 -1.3885370000 -3.4872550000

O -5.5401940000 -1.5186120000 -3.6298130000

O -4.2132360000 -3.8290270000 -2.7241470000

O -1.5256630000 -3.4018840000 -1.9085100000

O -2.0335820000 -0.6183480000 -4.2012010000

O 0.8415490000 -0.9259900000 -2.3906940000

H -4.0579840000 -2.1408390000 -4.9154630000

H -3.7784620000 -2.0797690000 -1.8754970000

H -1.8555130000 -3.2769840000 -3.9461710000

H -1.5330130000 -0.8019740000 -2.1954150000

H 0.3901590000 -2.4055100000 -3.7675010000

H 0.2453310000 -0.7380290000 -4.3587370000

C 8.0837990000 -5.6573250000 0.0578750000

H 7.7445270000 -6.6097760000 -0.3736460000

H 8.6083780000 -5.0861910000 -0.7242500000

H 8.7756120000 -5.8623000000 0.8769600000

C 4.5089850000 -4.0139670000 -3.4328920000

H 5.2381760000 -3.2514440000 -3.1517820000

H 4.9547790000 -5.0161280000 -3.3509900000

H 4.2092420000 -3.8543000000 -4.4804320000

C 2.2213760000 -0.8646940000 -2.7035800000

H 2.7473910000 -0.4834110000 -1.8262950000

H 2.6314320000 -1.8529810000 -2.9332040000

H 2.4060960000 -0.1770290000 -3.5398100000

C -6.2720860000 -0.7454780000 -4.5539130000

H -6.0570750000 -1.0441190000 -5.5907530000

H -7.3279970000 -0.9220830000 -4.3429160000

H -6.0695030000 0.3269250000 -4.4379380000

C -4.5427060000 -4.4909730000 -3.9251400000

H -5.3679960000 -3.9913160000 -4.4452530000

H -3.6854040000 -4.5819100000 -4.6088490000

H -4.8635900000 -5.4966240000 -3.6437760000

C -3.5015310000 -6.1540070000 -0.4977340000

H -3.2091520000 -7.2121190000 -0.5489700000

H -4.2960860000 -6.0336340000 0.2407990000

H -3.8711690000 -5.8190920000 -1.4723200000

C -0.6547720000 -5.5682860000 2.4008530000

H -1.7237630000 -5.7658310000 2.2734590000

H -0.2562850000 -6.2054420000 3.1944970000

H -0.5173730000 -4.5165170000 2.6735560000

C 1.2451270000 -2.8111540000 3.9159700000

H 0.2059290000 -2.4727460000 3.9400380000

H 1.3121940000 -3.7702910000 4.4480820000

H 1.8947330000 -2.0808970000 4.4098960000

C 4.6760860000 -0.3814860000 3.0612810000

H 4.3954320000 0.1939460000 2.1624740000

H 4.3876660000 0.1845090000 3.9514300000

H 5.7611650000 -0.5206620000 3.0491540000

C -3.0892540000 4.2933500000 -1.7995680000

C -4.4992010000 3.7716410000 -1.5391480000

C -4.5255010000 2.2510810000 -1.6956730000

C -3.8909920000 1.8069390000 -3.0186690000

C -2.6041140000 2.5781870000 -3.3656330000

C -2.1715410000 2.4415490000 -4.8288390000

O -4.8786930000 4.1822700000 -0.2502280000

O -5.8614450000 1.8223240000 -1.6076690000

O -3.6264210000 0.4143600000 -2.8615010000

O -2.7351010000 3.9769980000 -3.1218810000

O -3.2214150000 2.4439570000 -5.7635530000

H -3.0531660000 5.3816460000 -1.7159210000

H -5.1579860000 4.2244300000 -2.2946990000

H -3.9313390000 1.8212620000 -0.8787500000

H -4.6320030000 1.9536790000 -3.8133790000

H -1.8135580000 2.1790210000 -2.7211470000

H -1.4659330000 3.2599650000 -5.0420940000

H -1.6557760000 1.4889890000 -4.9630070000

C 1.4821300000 4.7421210000 0.7831470000

C 0.4655230000 4.0316240000 1.6809680000

C -0.7799690000 3.5223790000 0.9489400000

C -1.2936090000 4.4758590000 -0.1408050000

C -0.0993900000 4.9625640000 -0.9772680000

C -0.4446810000 5.9543150000 -2.0843470000

O 1.1549310000 2.9705550000 2.3308450000

O -1.6952840000 3.2544190000 1.9794330000

O -2.1926630000 3.6861660000 -0.9006880000

O 0.8482040000 5.5965030000 -0.1180040000

O -1.3572900000 6.9575270000 -1.7193540000

H 2.1370850000 5.3796300000 1.3745100000

H 0.1251470000 4.7604390000 2.4271580000

H -0.5215970000 2.5941410000 0.4163180000

H -1.8147210000 5.3287610000 0.3096800000

H 0.3715260000 4.0847260000 -1.4412870000

H 0.4991830000 6.3931150000 -2.4445060000

H -0.9147110000 5.4087350000 -2.9061900000

C 6.1376760000 3.2688750000 -1.0331920000

C 5.9500980000 4.4012210000 -0.0115900000

C 4.5753750000 4.2637620000 0.6453260000

C 3.4810010000 4.1745080000 -0.4217890000

C 3.8394760000 3.1255130000 -1.4821420000

C 2.8579490000 3.0579800000 -2.6399230000

O 6.9057180000 4.3812830000 1.0086780000

O 4.3127780000 5.3667370000 1.4810040000

O 2.2448550000 3.7508270000 0.1334990000

O 5.1251990000 3.3727870000 -2.0197160000

O 1.7888200000 2.2085880000 -2.2740280000

H 7.0823000000 3.3534390000 -1.5792310000

H 5.9866630000 5.3578180000 -0.5568530000

H 4.5616670000 3.3316410000 1.2275660000

H 3.3606360000 5.1545410000 -0.9025000000

H 3.8463630000 2.1359850000 -1.0085950000

H 3.3807320000 2.6450940000 -3.5146940000

H 2.4784820000 4.0567650000 -2.9033260000

C 7.0415150000 -1.7551160000 0.2059950000

C 8.0018600000 -0.7962160000 0.9156000000

C 7.4972260000 0.6374690000 0.8180100000

C 6.9721250000 1.0056850000 -0.5713290000

C 6.2407480000 -0.1382650000 -1.2973660000

C 6.1141890000 0.1403860000 -2.7801000000

O 8.1342480000 -1.1061630000 2.2816800000

O 8.5032440000 1.5694080000 1.1359110000

O 6.0512400000 2.0641410000 -0.3490160000

O 6.9214150000 -1.3720380000 -1.1439030000

O 5.1771300000 -0.7493960000 -3.3349570000

H 7.4508840000 -2.7642340000 0.2086270000

H 8.9767760000 -0.8640110000 0.4081690000

H 6.6501200000 0.7426110000 1.5057670000

H 7.8208490000 1.3552730000 -1.1750910000

H 5.2336890000 -0.2189210000 -0.8695020000

H 5.7860550000 1.1815300000 -2.9273940000

H 7.0995080000 0.0172950000 -3.2577420000

O 5.7905890000 -1.7060380000 0.8281840000

C -0.8323790000 7.9832580000 -0.9008140000

H 0.0905540000 8.3964870000 -1.3307910000

H -1.5909750000 8.7670220000 -0.8586380000

H -0.6136090000 7.6305570000 0.1133990000

C 0.8267450000 2.0929880000 -3.2990570000

H 1.2914900000 1.7758900000 -4.2434450000

H 0.1166680000 1.3326310000 -2.9784680000

H 0.3120350000 3.0496120000 -3.4621320000

C 5.0280240000 -0.5771330000 -4.7209720000

H 4.6787200000 0.4386110000 -4.9664820000

H 5.9714620000 -0.7583190000 -5.2562800000

H 4.2819650000 -1.3007850000 -5.0568400000

C -3.8347060000 3.7016700000 -5.9607550000

H -4.4914670000 3.5974660000 -6.8265490000

H -4.4240420000 4.0160070000 -5.0906530000

H -3.0845260000 4.4780600000 -6.1648940000

C 0.6980070000 2.6217390000 3.6326610000

H 1.4010440000 1.8771270000 4.0128550000

H 0.7141710000 3.4999120000 4.2887910000

H -0.3135020000 2.2122770000 3.6045060000

C -2.8534820000 2.5011560000 1.6550590000

H -2.6133040000 1.6889860000 0.9589720000

H -3.2096580000 2.0713750000 2.5953360000

H -3.6311410000 3.1252800000 1.2101500000

C -6.2523150000 4.4859930000 -0.0955760000

H -6.8791720000 3.6180470000 -0.3169970000

H -6.3856610000 4.7863770000 0.9455270000

H -6.5452740000 5.3214060000 -0.7474740000

C -6.0484910000 0.5773820000 -0.9548130000

H -5.5288960000 0.5659040000 0.0140280000

H -7.1232010000 0.4793830000 -0.7827420000

H -5.6915790000 -0.2627660000 -1.5543220000

C 8.8599920000 -2.2896250000 2.5474220000

H 8.3139330000 -3.1903470000 2.2366440000

H 9.8373190000 -2.2737440000 2.0444220000

H 9.0160230000 -2.3259740000 3.6268130000

C 8.5084290000 1.9821000000 2.4890760000

H 7.5805680000 2.5177970000 2.7301440000

H 8.6385070000 1.1320950000 3.1656750000

H 9.3516020000 2.6674100000 2.6002400000

C 8.2067590000 4.7566880000 0.6075500000

H 8.7708170000 4.9538170000 1.5207480000

H 8.7072270000 3.9504780000 0.0576910000

H 8.1866050000 5.6712720000 -0.0016180000

C 4.5441480000 5.1248330000 2.8558820000

H 4.3279310000 6.0603510000 3.3753690000

H 3.8704350000 4.3393290000 3.2307040000

H 5.5837120000 4.8365670000 3.0376950000

C -3.4051050000 -0.3432490000 -4.0108690000

H -3.6991860000 0.2178770000 -4.9029580000

C 5.1696960000 -2.8760150000 1.3452190000

H 5.7894120000 -3.3247020000 2.1297080000

C -4.4828640000 -2.5351000000 1.9078870000

H -4.9885980000 -3.2582060000 2.5547360000

C -2.9777240000 -2.5894530000 2.1770860000

C -5.2459190000 -1.2347260000 2.1150140000

H -2.7489680000 -3.6585410000 2.2172330000

H -2.4751280000 -2.2278030000 1.2783730000

C -2.4010270000 -1.9039190000 3.4251700000

O -6.4725110000 -1.2119930000 1.9844500000

H -3.1977580000 -1.6262330000 4.1195470000

H -1.7914060000 -2.6385590000 3.9612520000

C -1.5240030000 -0.6744360000 3.1308960000

H -2.1355560000 0.2119770000 2.9528250000

H -0.9165930000 -0.4550950000 4.0188320000

C -0.6104490000 -0.8768290000 1.9198130000

H -0.2502170000 -1.9013460000 1.8267500000

H -1.1066430000 -0.6052520000 0.9881420000

N 0.5978370000 -0.0232720000 2.0179720000

H 1.2672340000 -0.1305510000 1.2327670000

H 1.1238250000 -0.2072180000 2.8773450000

O -4.5554450000 -0.1515800000 2.3591450000

H -5.1430520000 0.6743650000 2.3230460000

N -4.6666340000 -2.8394350000 0.4772340000

H -3.9712120000 -3.5334090000 0.2017440000

H -5.5717700000 -3.2738830000 0.3085890000

H 0.3604890000 0.9745210000 2.0103460000

O -5.8784440000 2.1153000000 2.1500430000

H -5.5798410000 2.4283660000 1.2817230000

H -6.8276210000 1.8587770000 2.0108330000

O -6.6909810000 -4.2312140000 -1.4027640000

H -7.1608280000 -3.4169770000 -1.1302850000

H -5.8713280000 -3.9119640000 -1.8227840000

O -8.0740410000 -2.0862020000 -0.2142220000

H -8.6988930000 -2.6661990000 0.2435180000

H -7.4362360000 -1.8525490000 0.4837450000

O -8.2867720000 0.9771140000 1.7758570000

H -8.5464750000 1.1359050000 0.8573380000

H -7.7873570000 0.1391470000 1.7413670000

O -6.6754350000 0.4959020000 4.8763840000

H -6.8872370000 0.4605270000 3.9337960000

H -5.7764250000 0.8856920000 4.8994940000

O -5.6594270000 -2.0954590000 5.1220670000

H -6.0729600000 -1.2093480000 5.0348060000

H -4.8884400000 -1.9294060000 5.7032190000

O 2.5048760000 0.7377570000 0.2806790000

H 3.4378460000 0.5309740000 0.4236250000

H 2.4062550000 1.6732840000 0.5361890000

O -3.9599600000 1.0366270000 4.9677230000

H -3.8669970000 0.5595960000 4.1279150000

H -3.4148000000 1.8510180000 4.8607200000

O -2.5779630000 3.4531650000 4.7037070000

H -2.0475310000 3.4911320000 3.8877550000

H -3.4499540000 3.8303720000 4.4163140000

O -4.9297260000 4.3489180000 3.6596240000

H -5.2709500000 3.5581630000 3.1969110000

H -4.5363690000 4.8945490000 2.9412630000

O -3.7787830000 5.8947570000 1.6560770000

H -4.3702600000 6.6561620000 1.5728890000

H -4.0100220000 5.3394980000 0.8844040000

O -7.4072260000 -3.5098600000 3.4496080000

H -6.7672690000 -3.1952880000 4.1207730000

H -7.3198120000 -2.8294230000 2.7594220000

O -3.0441660000 -0.5040350000 -0.2534280000

H -3.7247790000 -1.2001590000 -0.1340760000

H -3.2233250000 -0.1491020000 -1.1404670000

O -6.1340870000 -5.5705190000 2.0746320000

H -6.5831250000 -4.8608810000 2.5860340000

H -5.2277110000 -5.5526830000 2.4082150000

O -0.9878910000 1.4949690000 6.1517960000

H -1.5754300000 0.7491460000 5.9716570000

H -1.4776070000 2.2476030000 5.7765860000

O -3.5234410000 -1.0974890000 6.6753950000

H -3.6210230000 -0.2781560000 6.1422360000

H -2.7153080000 -1.4971140000 6.3255660000

Cartesian coordinates of Complex A-1 (solution phase)

H -2.7973400000 5.3859620000 1.2212450000

C -0.7396960000 5.7955820000 -0.1848410000

C -0.3168270000 5.8359700000 -1.6535280000

C 0.9502300000 5.0193390000 -1.9187110000

C 1.4050460000 5.1665130000 0.5938850000

C 2.3739500000 5.5362520000 1.7108610000

O -1.3883900000 5.4047370000 -2.4509500000

O 1.4265730000 5.4825890000 -3.1633010000

O 0.3873310000 6.1649340000 0.5805810000

O 3.3596070000 6.4249700000 1.2404520000

H -1.4904020000 6.5694110000 0.0070880000

H -0.0746590000 6.8865450000 -1.8675050000

H 0.6609980000 3.9579100000 -1.9930490000

H 0.9581380000 4.1990280000 0.8478740000

H 1.7896730000 5.9911930000 2.5197720000

H 2.8356330000 4.6246510000 2.1159390000

C -3.4056380000 2.6674930000 3.1707100000

C -4.1606470000 3.0338630000 1.8959730000

C -3.1950340000 3.3301150000 0.7553260000

C -2.2301750000 4.4446860000 1.1742600000

C -1.5848760000 4.1347960000 2.5466070000

C -0.9577750000 5.3534940000 3.2053900000

O -5.0425570000 1.9878090000 1.5538920000

O -3.9586240000 3.6974680000 -0.3631620000

O -1.2391220000 4.5392210000 0.1513150000

O -2.5301960000 3.6905590000 3.5178940000

O 0.0519510000 4.9209800000 4.0963070000

H -4.0945470000 2.5938630000 4.0078270000

H -4.7273680000 3.9503060000 2.1105630000

H -2.6100740000 2.4218790000 0.5496070000

H -0.8289140000 3.3607220000 2.3724680000

H -0.5327900000 6.0405700000 2.4684280000

H -1.7560490000 5.8735790000 3.7547360000

C -4.8379560000 -1.1846090000 4.7343000000

C -4.4336590000 0.2331720000 4.3270450000

C -2.9816860000 0.3276830000 3.8135850000

C -2.5918050000 -0.8842280000 2.9347820000

C -1.0755200000 -0.9912890000 2.8463010000

O -6.2033240000 -1.1189350000 5.0376130000

O -4.6930380000 1.1772200000 5.3492820000

O -2.7355780000 1.4397760000 2.9656060000

O -3.0795930000 -2.1167470000 3.4420000000

O -0.6290620000 -2.0887320000 2.0394960000

H -4.2662850000 -1.5253330000 5.6131680000

H -5.0840240000 0.5240820000 3.4966500000

H -2.3099750000 0.3759800000 4.6852240000

H -3.0128690000 -0.7035600000 1.9394990000

H -0.6919860000 -0.0678650000 2.4054970000

H -0.6499520000 -1.0967120000 3.8540420000

C 4.1710230000 6.9163110000 2.2769110000

H 3.5780950000 7.4650950000 3.0226020000

H 4.7080370000 6.1028500000 2.7911560000

H 4.8970760000 7.5953260000 1.8259800000

C 0.4412820000 5.9281530000 4.9943200000

H 1.2392740000 5.5185810000 5.6179860000

H 0.8206630000 6.8190570000 4.4689340000

H -0.3961350000 6.2357290000 5.6368690000

C -0.4336060000 -3.2895610000 2.7779240000

H -1.3412750000 -3.5754950000 3.3117840000

H -0.1774040000 -4.0722610000 2.0570510000

H 0.3918240000 -3.1756710000 3.4946770000

C -6.8097290000 -2.3474700000 5.3681460000

H -6.2005700000 -2.9225620000 6.0807500000

H -7.7665160000 -2.1066960000 5.8353300000

H -7.0030450000 -2.9561080000 4.4750060000

C -4.2817120000 0.8518380000 6.6562940000

H -4.9268740000 0.0820250000 7.0972420000

H -3.2354540000 0.5168290000 6.7029160000

H -4.3758770000 1.7680760000 7.2416330000

C -6.3771810000 2.4167250000 1.3881840000

H -6.4504040000 3.1776230000 0.6021610000

H -6.9574620000 1.5391540000 1.0976090000

H -6.7881280000 2.8160340000 2.3263130000

C -3.5483050000 3.1262750000 -1.5899800000

H -3.3732050000 2.0471050000 -1.4857390000

H -4.3728310000 3.2684890000 -2.2912980000

H -2.6441190000 3.6081410000 -1.9741070000

C -1.5191120000 6.0449780000 -3.7025850000

H -2.4367120000 5.6558990000 -4.1508120000

H -1.6175130000 7.1330430000 -3.5841770000

H -0.6660080000 5.8362950000 -4.3566010000

C 2.4410080000 4.7162640000 -3.7818150000

H 2.1980110000 3.6468410000 -3.7578820000

H 2.4892590000 5.0584910000 -4.8193640000

H 3.4178600000 4.8523480000 -3.3056280000

C -6.3299710000 -2.9832570000 -1.1818130000

C -7.4964730000 -2.6220790000 -0.2674880000

C -6.9838060000 -1.9529340000 1.0048840000

C -5.8026080000 -2.6962190000 1.6314100000

C -4.8098980000 -3.2757520000 0.6137510000

C -3.8916740000 -4.3325140000 1.2322470000

O -8.3664360000 -1.7758080000 -0.9768480000

O -8.0343610000 -1.8976480000 1.9495360000

O -5.1630040000 -1.7406170000 2.4623430000

O -5.4822300000 -3.8780490000 -0.4872540000

O -4.4875340000 -5.1755380000 2.1851210000

H -6.6802840000 -3.5162500000 -2.0696090000

H -8.0015240000 -3.5617190000 0.0000220000

H -6.6418640000 -0.9449730000 0.7390690000

H -6.2097980000 -3.5088610000 2.2434380000

H -4.1860270000 -2.4504160000 0.2447540000

H -3.4702290000 -4.9315670000 0.4108340000

H -3.0845580000 -3.8163580000 1.7564070000

C -2.9243260000 -0.7853980000 -4.4423250000

C -4.0935190000 0.1723750000 -4.2476150000

C -4.8392680000 -0.1300030000 -2.9497060000

C -5.2266400000 -1.6137510000 -2.8706290000

C -4.0278340000 -2.5175650000 -3.2212970000

C -4.3763890000 -3.9967110000 -3.4077480000

O -3.5984050000 1.4843140000 -4.2761080000

O -5.9489320000 0.7232430000 -2.9195820000

O -5.6430380000 -1.8157830000 -1.5290040000

O -3.3802550000 -2.1119270000 -4.4289140000

O -5.5739740000 -4.2420400000 -4.0978400000

H -2.4763110000 -0.6283720000 -5.4270390000

H -4.7822380000 0.0007010000 -5.0884200000

H -4.1667740000 0.0832570000 -2.1051060000

H -6.0622860000 -1.8049780000 -3.5541740000

H -3.3179120000 -2.4304670000 -2.3853740000

H -3.5269810000 -4.4758740000 -3.9220440000

H -4.4990850000 -4.4601890000 -2.4270140000

C 2.1034320000 -0.4900440000 -2.8023330000

C 1.6079410000 0.2419450000 -4.0576060000

C 0.1162210000 0.5418560000 -3.9111010000

C -0.6215200000 -0.7866270000 -3.7911620000

C -0.0091050000 -1.6785120000 -2.7014290000

C -0.4267400000 -3.1307440000 -2.8737750000

O 2.3684970000 1.3978920000 -4.2848580000

O -0.3803880000 1.2365560000 -5.0256870000

O -1.9692810000 -0.5631030000 -3.4365710000

O 1.4225140000 -1.7155190000 -2.7059150000

O -0.2334490000 -3.8884190000 -1.6828950000

H 3.1635440000 -0.7337600000 -2.8836550000

H 1.7519540000 -0.4622240000 -4.8916240000

H -0.0439110000 1.1199320000 -2.9923930000

H -0.5600950000 -1.3022790000 -4.7610760000

H -0.3729370000 -1.2853740000 -1.7393100000

H 0.2111420000 -3.5805380000 -3.6393240000

H -1.4664430000 -3.1938110000 -3.2039470000

C 3.7619360000 3.4949610000 -0.1652540000

C 4.7538360000 2.6426320000 -0.9632660000

C 3.9955800000 1.5429970000 -1.7155240000

C 2.9314110000 0.8346880000 -0.8648600000

C 2.2191670000 1.7479490000 0.1448230000

C 1.5340730000 0.9541460000 1.2432030000

O 5.4539230000 3.3838900000 -1.9289930000

O 4.8688290000 0.5117180000 -2.1592260000

O 1.8577220000 0.2525120000 -1.6213330000

O 3.1287150000 2.6328430000 0.7698100000

O 0.7711860000 1.7720480000 2.0899620000

H 4.2532230000 4.2690390000 0.4303230000

H 5.4596660000 2.1866760000 -0.2522870000

H 3.5041760000 2.0110130000 -2.5720740000

H 3.4536940000 0.0417430000 -0.3174340000

H 1.4537130000 2.3125680000 -0.3963090000

H 0.8527820000 0.2374150000 0.7673840000

H 2.2856310000 0.3999440000 1.8195510000

O 2.8750450000 4.0168500000 -1.0833820000

C -5.5293540000 -3.9813360000 -5.4854000000

H -4.6686210000 -4.4799350000 -5.9528830000

H -6.4521790000 -4.3825410000 -5.9088270000

H -5.4654960000 -2.9085740000 -5.7029680000

C -1.4002050000 -4.0213670000 -0.8900900000

H -2.2150640000 -4.4643770000 -1.4736680000

H -1.1492900000 -4.6970520000 -0.0695970000

H -1.7322890000 -3.0588680000 -0.4749420000

C 1.3633230000 2.0085380000 3.3553530000

H 1.4034460000 1.0782110000 3.9422710000

H 2.3768440000 2.4108060000 3.2455650000

H 0.7414300000 2.7436790000 3.8671250000

C -5.3729790000 -6.1335430000 1.6404430000

H -5.6737810000 -6.7865190000 2.4618590000

H -6.2625480000 -5.6690030000 1.1981130000

H -4.8767080000 -6.7321900000 0.8635260000

C -4.4036060000 2.4079260000 -4.9791390000

H -3.8844810000 3.3681360000 -4.9299660000

H -4.5107880000 2.1159610000 -6.0342490000

H -5.3948020000 2.4990460000 -4.5260630000

C -6.4479550000 1.0567570000 -1.6362890000

H -5.6281470000 1.2041980000 -0.9207880000

H -6.9865970000 2.0013290000 -1.7505160000

H -7.1309610000 0.2871690000 -1.2640740000

C -9.7396830000 -1.9775720000 -0.7077650000

H -9.9689200000 -1.8268040000 0.3518210000

H -10.2854410000 -1.2491080000 -1.3108330000

H -10.0558160000 -2.9891980000 -1.0001420000

C -8.2843110000 -0.6173500000 2.4915940000

H -8.6214280000 0.0803780000 1.7112500000

H -9.0862180000 -0.7409330000 3.2227490000

H -7.4020160000 -0.2172500000 3.0010650000

C 6.3809690000 4.3046990000 -1.3936130000

H 5.8821680000 5.1580680000 -0.9169150000

H 7.0454720000 3.8202060000 -0.6630480000

H 6.9782140000 4.6716090000 -2.2298520000

C 5.6122030000 0.7981070000 -3.3348660000

H 4.9741900000 1.2653600000 -4.0919640000

H 6.4544350000 1.4607910000 -3.1232800000

H 5.9766820000 -0.1626600000 -3.7103230000

C 2.6325230000 1.6613680000 -5.6531740000

H 3.2077430000 2.5883580000 -5.6858040000

H 3.2308290000 0.8526120000 -6.0968790000

H 1.7032690000 1.7781180000 -6.2162430000

C -0.7388910000 2.5825980000 -4.7541340000

H -1.1532110000 2.9814440000 -5.6820760000

H -1.5021300000 2.6318970000 -3.9713630000

H 0.1326990000 3.1798020000 -4.4599120000

C -4.4848470000 -2.1509030000 3.6029070000

H -4.7152550000 -3.1919270000 3.8484610000

C 2.0213740000 5.1310130000 -0.8153360000

H 2.5846580000 6.0576760000 -0.9626850000

C 5.6554580000 -2.5726200000 1.7252250000

H 5.3518550000 -2.6635140000 0.6761660000

C 4.5199190000 -3.0584430000 2.6506340000

C 5.9420990000 -1.1056990000 1.9684500000

H 4.5526110000 -4.1528680000 2.6065840000

H 4.7764470000 -2.7825860000 3.6794400000

C 3.0968580000 -2.5803790000 2.3502820000

O 6.9425620000 -0.6900680000 2.5460570000

H 2.4273100000 -3.1821630000 2.9729900000

H 2.9618960000 -1.5424190000 2.6680270000

C 2.6665710000 -2.6879570000 0.8894550000

H 1.6099110000 -2.4125160000 0.7987070000

H 3.2303910000 -1.9701990000 0.2827070000

C 2.8747560000 -4.0768340000 0.3066440000

H 3.9085490000 -4.4147460000 0.3995350000

H 2.2267760000 -4.8202370000 0.7761180000

N 2.5682690000 -4.0480280000 -1.1426050000

H 2.8648630000 -4.9085900000 -1.6351640000

H 3.1117030000 -3.2846840000 -1.5851800000

O 4.9905180000 -0.3193240000 1.5165830000

H 5.0939560000 0.6057820000 1.8989810000

N 6.8146200000 -3.4255940000 1.9271780000

H 7.6089630000 -3.0621770000 1.4044640000

H 7.0684110000 -3.4406010000 2.9135510000

H 1.5592200000 -3.8934870000 -1.3056200000

O 7.2678710000 0.3123410000 -0.6408000000

H 6.3725560000 0.2426790000 -1.0269660000

H 7.8239460000 0.6299580000 -1.3667170000

O 5.0827200000 2.0487160000 2.6538410000

H 4.3786030000 2.3871500000 2.0662910000

H 5.9478970000 2.2913320000 2.2423260000

O 7.5775270000 1.9289870000 1.6072620000

H 7.5469890000 1.1137800000 2.1430380000

H 7.4383230000 1.5581320000 0.7104210000

O 8.9416190000 -1.6577020000 0.3163190000

H 8.2415460000 -1.0538390000 -0.0134810000

H 8.9731590000 -1.4741970000 1.2652890000

O 6.0557650000 -5.8709940000 0.9142530000

H 6.9327540000 -6.2662980000 0.7142210000

H 6.3283110000 -4.9921610000 1.3086600000

O 3.8849100000 -5.6360010000 -3.0000480000

H 4.7505400000 -5.3942820000 -2.5928940000

H 3.9477110000 -6.5756510000 -3.2159860000

O 6.0602260000 -4.6945710000 -1.5954720000

H 5.6457030000 -3.8036580000 -1.5646610000

H 5.9073290000 -5.0973070000 -0.7082570000

O 6.6008090000 -1.5535460000 5.1956580000

H 7.0365520000 -1.4116120000 4.3380750000

H 5.7629810000 -1.0531540000 5.0781380000

O 4.4074390000 0.1479570000 4.7066760000

H 4.7253470000 0.8154060000 4.0689030000

H 3.6922040000 -0.2947730000 4.2306440000

O 8.7589320000 -4.1923410000 -0.8531340000

H 7.8320630000 -4.2958650000 -1.1531830000

H 8.7855370000 -3.3188670000 -0.4111480000

O 4.7089310000 -2.3528090000 -2.0229050000

H 4.7773740000 -2.2452920000 -3.0036740000

H 4.8163940000 -1.4470520000 -1.6855150000

O 6.5786430000 -4.3085920000 5.1565570000

H 5.6935370000 -4.5534380000 4.8543390000

H 6.5548370000 -3.3227250000 5.1719540000

O 4.6975330000 -2.0088530000 -4.7741180000

H 4.2751840000 -1.1447670000 -4.8834490000

H 3.9456860000 -2.6456980000 -4.8472730000

O 2.5850840000 -3.6772630000 -4.5492130000

H 2.2207550000 -3.0679380000 -3.8802270000

H 3.0319520000 -4.3830060000 -4.0334370000

O 7.6821410000 -6.2168330000 3.4445590000

H 6.8996970000 -6.3025210000 2.8764990000

H 7.4571950000 -5.4492910000 4.0113400000

O 8.8016270000 -6.3741170000 0.8302490000

H 8.6679030000 -6.1197300000 1.7635800000

H 8.8526750000 -5.5243270000 0.3336880000

Cartesian coordinates of Complex A-2 (solution phase)

H -2.2294780000 5.6965610000 -0.2293280000

C -0.1664590000 5.5288700000 -1.6772980000

C 0.2337180000 5.1420520000 -3.1012760000

C 1.4160340000 4.1705390000 -3.1317190000

C 1.9243610000 4.9306850000 -0.7431170000

C 2.9415980000 5.4897190000 0.2447240000

O -0.8859200000 4.6174360000 -3.7655230000

O 1.9120370000 4.2436350000 -4.4505190000

O 1.0023870000 5.9802630000 -1.0272340000

O 3.9965940000 6.1278310000 -0.4352830000

H -0.8402030000 6.3924950000 -1.7018830000

H 0.5670390000 6.0726810000 -3.5824120000

H 1.0293010000 3.1590260000 -2.9260730000

H 1.3947940000 4.1103070000 -0.2467530000

H 2.4147790000 6.1940360000 0.9005260000

H 3.3243380000 4.6785150000 0.8793610000

C -3.0512450000 3.6550130000 2.3623240000

C -3.7907250000 3.7415230000 1.0300720000

C -2.8212420000 3.6363660000 -0.1405210000

C -1.7514800000 4.7274030000 -0.0223170000

C -1.1144260000 4.7309870000 1.3883310000

C -0.3674070000 6.0164470000 1.7077470000

O -4.7703050000 2.7287760000 0.9685540000

O -3.5664950000 3.7656560000 -1.3224390000

O -0.7731030000 4.4570470000 -1.0260510000

O -2.0800510000 4.6475490000 2.4346560000

O 0.6132580000 5.7417420000 2.6891990000

H -3.7299990000 3.8685180000 3.1839280000

H -4.2676670000 4.7307500000 0.9913310000

H -2.3256160000 2.6560620000 -0.0948220000

H -0.4357170000 3.8719920000 1.4304890000

H 0.1060540000 6.4432860000 0.8194100000

H -1.1056440000 6.7346940000 2.0944430000

C -4.8040420000 0.5000270000 4.8740540000

C -4.2784450000 1.7168890000 4.1108940000

C -2.8327070000 1.5378800000 3.6023710000

C -2.5702210000 0.1067170000 3.0771060000

C -1.0718120000 -0.1600250000 3.0321570000

O -6.1524230000 0.7695680000 5.1382940000

O -4.4331370000 2.9164510000 4.8460850000

O -2.4999680000 2.3598750000 2.4933110000

O -3.1603460000 -0.8982120000 3.8871960000

O -0.7413380000 -1.4676310000 2.5466570000

H -4.2512010000 0.3509710000 5.8163440000

H -4.9132180000 1.8381500000 3.2282000000

H -2.1448620000 1.7513620000 4.4359570000

H -2.9895080000 0.0575920000 2.0661070000

H -0.6127000000 0.5754480000 2.3667180000

H -0.6407780000 -0.0354440000 4.0354310000

C 4.8667340000 6.7972100000 0.4415830000

H 4.3387890000 7.5758970000 1.0115790000

H 5.3352450000 6.1020600000 1.1562440000

H 5.6439310000 7.2639030000 -0.1664870000

C 1.1081180000 6.9091420000 3.2932400000

H 1.8753820000 6.6061710000 4.0092940000

H 1.5583980000 7.5916070000 2.5549100000

H 0.3130370000 7.4514610000 3.8254910000

C -0.6443460000 -2.4446010000 3.5769180000

H -1.5651000000 -2.4944030000 4.1598610000

H -0.4727660000 -3.4094160000 3.0896810000

H 0.2001430000 -2.2230270000 4.2448170000

C -6.8631640000 -0.2670180000 5.7760160000

H -6.2971710000 -0.6884740000 6.6200240000

H -7.7857440000 0.1756540000 6.1558110000

H -7.1261240000 -1.0686630000 5.0734780000

C -4.0315490000 2.9096710000 6.1959690000

H -4.7369290000 2.3461700000 6.8191940000

H -3.0196460000 2.5031690000 6.3377910000

H -4.0315540000 3.9522100000 6.5186900000

C -6.0626460000 3.2209070000 0.6850210000

H -6.0792200000 3.7517130000 -0.2739520000

H -6.7257740000 2.3555990000 0.6315790000

H -6.4197080000 3.8893110000 1.4817920000

C -3.2308600000 2.8563960000 -2.3520060000

H -3.1538260000 1.8315350000 -1.9655670000

H -4.0507920000 2.8852420000 -3.0724160000

H -2.2931770000 3.1345670000 -2.8420950000

C -0.9784550000 4.9152330000 -5.1425960000

H -1.9356570000 4.5092720000 -5.4796570000

H -0.9753000000 6.0008950000 -5.3156660000

H -0.1595690000 4.4642020000 -5.7115320000

C 2.8414250000 3.2510680000 -4.8370810000

H 2.5016150000 2.2535640000 -4.5342510000

H 2.9030540000 3.3029810000 -5.9277730000

H 3.8341030000 3.4170940000 -4.4058270000

C -6.5532330000 -2.6452070000 -0.3708880000

C -7.6662990000 -1.9496230000 0.4065520000

C -7.0732940000 -1.0198640000 1.4617320000

C -5.9548380000 -1.6783920000 2.2714400000

C -5.0365390000 -2.5947280000 1.4504600000

C -4.2087610000 -3.5320050000 2.3329030000

O -8.4668210000 -1.2428620000 -0.5077560000

O -8.0984470000 -0.6207840000 2.3498900000

O -5.2166150000 -0.6014630000 2.8263070000

O -5.7795590000 -3.4004200000 0.5416690000

O -4.8633920000 -4.0356190000 3.4694020000

H -6.9656280000 -3.3580120000 -1.0895640000

H -8.2506430000 -2.7347720000 0.9082930000

H -6.6449570000 -0.1535290000 0.9426940000

H -6.4242860000 -2.2600260000 3.0727900000

H -4.3458700000 -1.9569630000 0.8821190000

H -3.8575340000 -4.3623410000 1.7017200000

H -3.3489130000 -2.9732000000 2.7089920000

C -3.0155780000 -1.7082690000 -4.0677810000

C -4.0887060000 -0.6288350000 -4.1417210000

C -4.8372140000 -0.5085830000 -2.8160450000

C -5.3574870000 -1.8766980000 -2.3519370000

C -4.2525400000 -2.9480900000 -2.4424210000

C -4.7381840000 -4.3852410000 -2.2353500000

O -3.4759850000 0.5775070000 -4.5109210000

O -5.8633470000 0.4215970000 -3.0207760000

O -5.7681490000 -1.6791730000 -1.0079120000

O -3.5907940000 -2.9364090000 -3.7089870000

O -5.9645880000 -4.6913380000 -2.8460270000

H -2.5717460000 -1.8576620000 -5.0554620000

H -4.8044080000 -0.9506760000 -4.9129970000

H -4.1341020000 -0.1437240000 -2.0519980000

H -6.2185130000 -2.1624470000 -2.9675300000

H -3.5237360000 -2.7101310000 -1.6533170000

H -3.9448750000 -5.0591970000 -2.5984530000

H -4.8862920000 -4.5607450000 -1.1681980000

C 2.0448050000 -1.4595820000 -2.5231830000

C 1.5975300000 -1.0409180000 -3.9309070000

C 0.1422110000 -0.5759950000 -3.8806040000

C -0.7120030000 -1.7517560000 -3.4208060000

C -0.1657250000 -2.3783660000 -2.1296960000

C -0.7174560000 -3.7795580000 -1.9166690000

O 2.4569160000 -0.0613550000 -4.4485360000

O -0.3071800000 -0.1561650000 -5.1427870000

O -2.0274790000 -1.3189010000 -3.1484840000

O 1.2562060000 -2.5480110000 -2.1127060000

O -0.5745210000 -4.2118700000 -0.5666750000

H 3.0765760000 -1.8127730000 -2.5291320000

H 1.6623460000 -1.9493100000 -4.5489270000

H 0.0509140000 0.2361510000 -3.1480440000

H -0.7143860000 -2.5073200000 -4.2201760000

H -0.4758950000 -1.7135900000 -1.3082180000

H -0.1362740000 -4.4716150000 -2.5317060000

H -1.7638890000 -3.8301640000 -2.2270270000

C 4.1051990000 2.9071580000 -1.0160210000

C 5.0013860000 1.7866990000 -1.5532510000

C 4.1331190000 0.6031490000 -1.9952830000

C 3.0229210000 0.2454640000 -0.9966260000

C 2.4142940000 1.4541720000 -0.2690080000

C 1.6778420000 1.0443750000 0.9941610000

O 5.7502420000 2.1793490000 -2.6744500000

O 4.9007160000 -0.5850920000 -2.1444430000

O 1.8879440000 -0.4131470000 -1.5815450000

O 3.4114510000 2.3838340000 0.1080460000

O 1.0073590000 2.1232530000 1.5893330000

H 4.6750010000 3.7621870000 -0.6416240000

H 5.6741460000 1.4710410000 -0.7418400000

H 3.6722210000 0.8734610000 -2.9489640000

H 3.4794310000 -0.4199220000 -0.2556410000

H 1.6946890000 1.9254840000 -0.9459810000

H 0.9258820000 0.2944920000 0.7184590000

H 2.3850100000 0.5944810000 1.7020650000

O 3.2546910000 3.2488950000 -2.0463000000

C -5.9194670000 -4.8101040000 -4.2528380000

H -5.1159380000 -5.4914400000 -4.5655440000

H -6.8821030000 -5.2209510000 -4.5629720000

H -5.7613210000 -3.8428090000 -4.7448210000

C -1.7350990000 -4.0226690000 0.2236940000

H -2.5966010000 -4.5257680000 -0.2291880000

H -1.5332400000 -4.4786980000 1.1950610000

H -1.9705320000 -2.9580900000 0.3677980000

C 1.6397240000 2.6284110000 2.7524140000

H 1.6042900000 1.8859950000 3.5639770000

H 2.6837910000 2.8921960000 2.5486160000

H 1.0963540000 3.5269900000 3.0474190000

C -5.8417760000 -5.0167470000 3.1892880000

H -6.1869920000 -5.3998690000 4.1512830000

H -6.6920620000 -4.6045260000 2.6331020000

H -5.4151660000 -5.8420280000 2.6015310000

C -4.2049050000 1.3542950000 -5.4388920000

H -3.5996240000 2.2415760000 -5.6401250000

H -4.3563350000 0.8067140000 -6.3805180000

H -5.1761590000 1.6532350000 -5.0336270000

C -6.3082510000 1.1258050000 -1.8746980000

H -5.4666970000 1.3798110000 -1.2166040000

H -6.7601180000 2.0529550000 -2.2378290000

H -7.0526740000 0.5481680000 -1.3181440000

C -9.8480920000 -1.2384430000 -0.2060110000

H -10.0447840000 -0.7937830000 0.7747980000

H -10.3348960000 -0.6469840000 -0.9837470000

H -10.2603570000 -2.2575810000 -0.2237710000

C -8.2209560000 0.7744530000 2.5334950000

H -8.5057860000 1.2704000000 1.5945280000

H -9.0184420000 0.9223690000 3.2652430000

H -7.2973140000 1.2108010000 2.9269020000

C 6.7664950000 3.1186390000 -2.3932110000

H 6.3556980000 4.1105280000 -2.1624090000

H 7.3957340000 2.7846700000 -1.5557660000

H 7.3804770000 3.1963890000 -3.2919890000

C 5.6474200000 -0.6881510000 -3.3480730000

H 5.0421430000 -0.3787370000 -4.2068780000

H 6.5500440000 -0.0734330000 -3.3121390000

H 5.9159170000 -1.7426610000 -3.4546390000

C 2.7211440000 -0.1924490000 -5.8356800000

H 3.3776910000 0.6362930000 -6.1070440000

H 3.2349710000 -1.1407940000 -6.0461150000

H 1.7967540000 -0.1416620000 -6.4176450000

C -0.5364590000 1.2412860000 -5.2382620000

H -0.9281620000 1.4191090000 -6.2418870000

H -1.2788930000 1.5658330000 -4.5023170000

H 0.3908060000 1.8118850000 -5.1049290000

C -4.5599280000 -0.7583720000 4.0400410000

H -4.8803350000 -1.6719130000 4.5495670000

C 2.5112730000 4.4686570000 -2.0881450000

H 3.1540600000 5.2680560000 -2.4699110000

C 5.5291370000 -2.4402630000 2.4919870000

H 5.2420730000 -2.7669380000 1.4858620000

C 4.3817360000 -2.7254860000 3.4832840000

C 5.8113930000 -0.9523740000 2.4039190000

H 4.3964120000 -3.8103020000 3.6346610000

H 4.6431640000 -2.2755460000 4.4493730000

C 2.9642080000 -2.2908310000 3.0948470000

O 6.8233670000 -0.4222420000 2.8550000000

H 2.2873700000 -2.7334420000 3.8342310000

H 2.8486790000 -1.2064380000 3.1880100000

C 2.5089220000 -2.7021250000 1.6940620000

H 1.4634940000 -2.4036580000 1.5552180000

H 3.0900040000 -2.1658980000 0.9354610000

C 2.6412790000 -4.1954940000 1.4414870000

H 3.6718630000 -4.5423480000 1.5487960000

H 2.0100470000 -4.7805750000 2.1142210000

N 2.2429800000 -4.5171510000 0.0482830000

H 2.4821650000 -5.5042160000 -0.1735510000

H 2.7978580000 -3.9253640000 -0.5919490000

O 4.8624240000 -0.2647370000 1.8122610000

H 5.0553030000 0.7203360000 1.8971840000

N 6.6963450000 -3.2024910000 2.9040310000

H 7.4612140000 -3.0359050000 2.2505470000

H 7.0072990000 -2.8796770000 3.8243890000

H 1.2395070000 -4.3436080000 -0.1168580000

O 7.2372590000 -0.6197520000 -0.4857680000

H 6.3544790000 -0.6897050000 -0.9007990000

H 7.8391620000 -0.5053030000 -1.2359650000

O 5.3234770000 2.3228920000 2.1077240000

H 4.6481260000 2.5512740000 1.4421710000

H 6.1914590000 2.2882430000 1.6349180000

O 7.7643670000 1.6238570000 1.1196720000

H 7.7488930000 1.0847590000 1.9295170000

H 7.5420280000 0.9501280000 0.4419930000

O 8.9588630000 -2.5206300000 0.7435000000

H 8.2549640000 -1.9320090000 0.4061340000

H 9.2329020000 -2.1215950000 1.5951500000

O 6.4880220000 -5.8830650000 2.3853460000

H 7.3510400000 -5.8980430000 1.9239940000

H 6.4682240000 -4.9364260000 2.7011180000

O 3.5064200000 -6.9626150000 -0.4961470000

H 4.3819570000 -6.5059240000 -0.4215600000

H 3.4952420000 -7.6269730000 0.2055760000

O 5.7654930000 -5.4926710000 -0.2208530000

H 5.4048940000 -4.6017650000 -0.4245870000

H 5.8222660000 -5.5507570000 0.7631380000

O 6.7730920000 0.5423060000 5.5235800000

H 7.1875350000 0.5620790000 4.6480140000

H 5.8347660000 0.7322880000 5.2992060000

O 4.2889770000 1.3288130000 4.5537530000

H 4.6913050000 1.7741620000 3.7820500000

H 3.8845790000 0.5419140000 4.1624210000

O 8.5264990000 -5.2811530000 0.4812650000

H 7.6916560000 -5.3177540000 -0.0226480000

H 8.6467100000 -4.3222060000 0.6504160000

O 4.4849280000 -3.2630420000 -1.1605600000

H 4.2686000000 -3.6164350000 -2.0611020000

H 4.6946720000 -2.3213350000 -1.2921440000

O -0.3093140000 -6.8924700000 -1.2258710000

H -1.0177450000 -6.9192520000 -1.8844460000

H -0.4046360000 -6.0014280000 -0.8324970000

O 3.6222550000 -4.3813070000 -3.4739930000

H 3.1336650000 -3.7182130000 -3.9792560000

H 2.9297280000 -5.0166960000 -3.1739270000

O 1.9912420000 -6.4663010000 -2.7512540000

H 1.1251890000 -6.5152990000 -2.2902330000

H 2.6065250000 -6.7916360000 -2.0643530000

O 7.7351280000 -2.1150790000 5.6252720000

H 7.2710670000 -1.2514810000 5.6366760000

H 8.4818530000 -1.9444050000 5.0229640000

O 9.3540150000 -1.4312740000 3.3183460000

H 8.4766840000 -1.0199710000 3.1750900000

H 9.9658230000 -0.6833120000 3.3673040000

Cartesian coordinates of Complex B-1 (solution phase)

H -1.0135040000 -5.4365170000 2.1470630000

C -2.0903380000 -4.7495240000 -0.0259430000

C -2.2083320000 -4.0305130000 -1.3690240000

C -3.3405980000 -3.0020930000 -1.3799570000

C -4.2939680000 -4.1988990000 0.6022890000

C -5.4495580000 -4.8529740000 1.3367310000

O -0.9709160000 -3.4245570000 -1.6726550000

O -3.4566270000 -2.6161600000 -2.7300730000

O -3.3441290000 -5.2330400000 0.3659440000

O -6.2778700000 -5.5473380000 0.4327050000

H -1.4478600000 -5.6241310000 -0.1282810000

H -2.4567240000 -4.7942960000 -2.1182220000

H -3.0369960000 -2.1362140000 -0.7670890000

H -3.8595530000 -3.4515010000 1.2748620000

H -5.0141710000 -5.5442520000 2.0728030000

H -6.0256480000 -4.0956510000 1.8891720000

C 1.5908920000 -3.5347480000 3.4466490000

C 1.8074190000 -4.5905480000 2.3552350000

C 0.7229070000 -4.5123260000 1.2872700000

C -0.6865710000 -4.4218570000 1.8813560000

C -0.7701620000 -3.5135020000 3.1135540000

C -1.9971120000 -3.7507330000 3.9900930000

O 3.0414580000 -4.4753160000 1.6997190000

O 0.7478780000 -5.6644130000 0.4702880000

O -1.5589580000 -3.8575350000 0.9146530000

O 0.3186090000 -3.7151060000 4.0114730000

O -3.1406130000 -3.2119780000 3.3823110000

H 2.2925750000 -3.6839080000 4.2725750000

H 1.7287880000 -5.5703290000 2.8516310000

H 0.8841400000 -3.6142950000 0.6795190000

H -0.7742710000 -2.4813630000 2.7467160000

H -2.1228950000 -4.8279920000 4.1838900000

H -1.8120850000 -3.2602000000 4.9591240000

C 3.9804150000 0.2980910000 4.5282680000

C 3.5996850000 -0.9229020000 3.6920430000

C 2.1042110000 -1.2306660000 3.8278060000

C 1.2728040000 0.0230740000 3.5320920000

C -0.2253780000 -0.1398780000 3.7904270000

O 5.3330590000 0.5554540000 4.2811570000

O 4.3793870000 -2.0725370000 3.9598390000

O 1.7463560000 -2.2510510000 2.9081450000

O 1.7066660000 1.0761340000 4.3882710000

O -0.9329870000 -0.0896140000 2.5683160000

H 3.8237730000 0.0894110000 5.6009530000

H 3.8191720000 -0.6827990000 2.6468340000

H 1.8705640000 -1.5551440000 4.8515320000

H 1.4177410000 0.3086470000 2.4817130000

H -0.4369470000 -1.0817460000 4.3170060000

H -0.5465610000 0.6855060000 4.4428480000

C -7.3048870000 -6.2559670000 1.0830080000

H -6.8956770000 -7.0063270000 1.7743750000

H -7.9632900000 -5.5796630000 1.6506510000

H -7.8892530000 -6.7614320000 0.3119460000

C -4.2867490000 -3.2790930000 4.1966010000

H -5.0726420000 -2.7253100000 3.6790210000

H -4.6006430000 -4.3199570000 4.3634400000

H -4.1055690000 -2.8120120000 5.1772600000

C -2.3331530000 -0.1416780000 2.7742580000

H -2.8180670000 -0.0791050000 1.7982240000

H -2.6419600000 -1.0831890000 3.2387840000

H -2.6744000000 0.7073690000 3.3816700000

C 5.8797630000 1.6366720000 5.0018870000

H 5.6075870000 1.5914230000 6.0669330000

H 6.9640670000 1.5600140000 4.9066670000

H 5.5624470000 2.6028360000 4.5891280000

C 4.6736860000 -2.3463040000 5.3117130000

H 5.3823750000 -1.6185970000 5.7233510000

H 3.7742010000 -2.3745220000 5.9448150000

H 5.1364760000 -3.3357210000 5.3312390000

C 4.1569180000 -4.9726320000 2.4079390000

H 3.9891490000 -6.0110120000 2.7265670000

H 4.9959140000 -4.9395060000 1.7106330000

H 4.3930350000 -4.3474840000 3.2750990000

C 1.5388260000 -5.5643820000 -0.6976320000

H 2.6074870000 -5.5710040000 -0.4611360000

H 1.2935660000 -6.4352450000 -1.3107960000

H 1.3027890000 -4.6496240000 -1.2517160000

C -0.5286890000 -3.5892710000 -3.0065980000

H 0.4606190000 -3.1275230000 -3.0622710000

H -0.4319570000 -4.6535430000 -3.2626240000

H -1.2125220000 -3.1106520000 -3.7154860000

C -4.2884480000 -1.5230510000 -3.0362410000

H -4.1618180000 -0.7002160000 -2.3118870000

H -3.9889220000 -1.1739870000 -4.0283580000

H -5.3456680000 -1.8039860000 -3.0484440000

C 2.3859840000 5.2903370000 0.8125110000

C 3.8659350000 4.9203690000 0.7831230000

C 4.0585910000 3.5120610000 1.3459750000

C 3.3627490000 3.3439160000 2.7015890000

C 1.9668250000 3.9919470000 2.7537000000

C 1.4211370000 4.1822200000 4.1724230000

O 4.3104930000 5.0299070000 -0.5451580000

O 5.4385080000 3.2655250000 1.4529870000

O 3.2814080000 1.9356800000 2.9118670000

O 1.9521520000 5.2831380000 2.1488330000

O 2.3730040000 4.5741210000 5.1296580000

H 2.2284060000 6.3032870000 0.4359710000

H 4.3939670000 5.6414670000 1.4242820000

H 3.5973390000 2.8059410000 0.6432830000

H 4.0049240000 3.7942470000 3.4677510000

H 1.2919310000 3.3326740000 2.1973250000

H 0.6065340000 4.9214290000 4.1162220000

H 1.0123310000 3.2352720000 4.5295590000

C -1.9498320000 4.4100040000 -2.0501960000

C -0.7777790000 3.6389210000 -2.6634800000

C 0.4470100000 3.5164830000 -1.7517040000

C 0.7412570000 4.7827160000 -0.9328730000

C -0.5741080000 5.2992660000 -0.3281490000

C -0.4524330000 6.5831720000 0.4874520000

O -1.2734050000 2.3599250000 -3.0404260000

O 1.4783860000 3.1194670000 -2.6178260000

O 1.6543720000 4.3513050000 0.0618380000

O -1.5075450000 5.5492270000 -1.3790370000

O 0.3638650000 7.5736580000 -0.0827260000

H -2.6189690000 4.7738870000 -2.8279770000

H -0.4595610000 4.1883130000 -3.5583250000

H 0.2531950000 2.7323390000 -1.0035770000

H 1.1962480000 5.5528180000 -1.5667900000

H -0.9777470000 4.5158490000 0.3283650000

H -1.4708700000 6.9647770000 0.6615250000

H 0.0017850000 6.3413910000 1.4514250000

C -6.5437730000 2.8272560000 -0.1702320000

C -6.3993830000 3.6691750000 -1.4475010000

C -4.9636200000 3.5581810000 -1.9638040000

C -3.9690010000 3.9031840000 -0.8520190000

C -4.2958270000 3.1271940000 0.4302700000

C -3.4236900000 3.5008300000 1.6169350000

O -7.2481220000 3.2513720000 -2.4768780000

O -4.7586230000 4.4309010000 -3.0501740000

O -2.6454910000 3.5259060000 -1.2011520000

O -5.6457230000 3.3244200000 0.8072390000

O -2.2319570000 2.7429540000 1.5553630000

H -7.5378480000 2.9187230000 0.2781770000

H -6.5994730000 4.7200190000 -1.1840180000

H -4.7856530000 2.5179130000 -2.2707350000

H -4.0108090000 4.9814550000 -0.6480180000

H -4.1413680000 2.0575160000 0.2421520000

H -3.9716560000 3.2613900000 2.5396350000

H -3.1916180000 4.5765180000 1.6216900000

C -6.7254470000 -2.4212410000 -0.0547990000

C -7.7239170000 -1.8236110000 -1.0503920000

C -7.4046710000 -0.3581840000 -1.3154270000

C -7.0568930000 0.4285370000 -0.0498400000

C -6.2642530000 -0.3734880000 0.9986080000

C -6.3084160000 0.2964920000 2.3555240000

O -7.6925140000 -2.4939210000 -2.2871070000

O -8.4810330000 0.3093320000 -1.9298390000

O -6.2521280000 1.5101290000 -0.4973730000

O -6.7762200000 -1.6869780000 1.1459010000

O -5.3269930000 -0.2786450000 3.1823830000

H -7.0097770000 -3.4430340000 0.1921700000

H -8.7262580000 -1.8919750000 -0.5996300000

H -6.5165850000 -0.3188910000 -1.9568450000

H -7.9930100000 0.8015930000 0.3878630000

H -5.2197950000 -0.4217670000 0.6661540000

H -6.1214870000 1.3752460000 2.2343370000

H -7.3117170000 0.1660820000 2.7920100000

O -5.4374820000 -2.3621960000 -0.5951650000

C -0.2020780000 8.2673950000 -1.1763940000

H -1.2031860000 8.6456630000 -0.9271180000

H 0.4584550000 9.1108440000 -1.3857320000

H -0.2832820000 7.6358400000 -2.0682650000

C -1.3611880000 3.0333220000 2.6263780000

H -1.8695560000 2.9116220000 3.5933750000

H -0.5391990000 2.3222970000 2.5639470000

H -0.9811750000 4.0614530000 2.5537480000

C -5.3273040000 0.2676420000 4.4764150000

H -5.1254370000 1.3506590000 4.4576850000

H -6.2875830000 0.1025820000 4.9864000000

H -4.5343920000 -0.2323930000 5.0370760000

C 2.8114820000 5.9124900000 5.0140720000

H 3.3937180000 6.1298460000 5.9115960000

H 3.4365540000 6.0682730000 4.1262560000

H 1.9586530000 6.6029660000 4.9579420000

C -0.6606180000 1.7512660000 -4.1714840000

H -1.2321340000 0.8429940000 -4.3755170000

H -0.7209410000 2.4171860000 -5.0403260000

H 0.3855550000 1.5083040000 -3.9764050000

C 2.6833220000 2.6456250000 -2.0368890000

H 2.4787020000 2.0174680000 -1.1619650000

H 3.1729520000 2.0396880000 -2.8042330000

H 3.3370480000 3.4659880000 -1.7337860000

C 5.6465600000 5.4710340000 -0.6979980000

H 6.3493060000 4.7867460000 -0.2151000000

H 5.8381780000 5.5054780000 -1.7722450000

H 5.7768880000 6.4797560000 -0.2806010000

C 5.8314970000 1.9321490000 1.1725090000

H 5.4081630000 1.5964850000 0.2150260000

H 6.9215100000 1.9433250000 1.0945830000

H 5.5236990000 1.2358690000 1.9555490000

C -8.2447200000 -3.7948550000 -2.2638360000

H -7.6261460000 -4.4984430000 -1.6907740000

H -9.2589940000 -3.7847680000 -1.8400810000

H -8.2955790000 -4.1324030000 -3.3002860000

C -8.4110980000 0.3505110000 -3.3422190000

H -7.5354350000 0.9287250000 -3.6660860000

H -8.3763950000 -0.6559010000 -3.7701820000

H -9.3160820000 0.8589090000 -3.6820580000

C -8.6158940000 3.5333480000 -2.2665470000

H -9.1131930000 3.4053330000 -3.2294870000

H -9.0651530000 2.8366030000 -1.5485640000

H -8.7607690000 4.5685890000 -1.9270750000

C -4.8323730000 3.8095300000 -4.3193440000

H -4.6823210000 4.5981440000 -5.0591920000

H -4.0383950000 3.0552460000 -4.4291300000

H -5.8091160000 3.3420280000 -4.4751150000

C 3.0473160000 1.4803470000 4.2087360000

H 3.1893660000 2.2897260000 4.9311800000

C -4.6368780000 -3.5281950000 -0.7407180000

H -5.1241410000 -4.2474570000 -1.4085850000

C 4.4202260000 -1.6434300000 -1.7650140000

H 4.8814230000 -0.8266390000 -1.1989560000

C 3.0215240000 -1.1888530000 -2.2316650000

C 4.2575070000 -2.8063740000 -0.8128840000

H 3.1750730000 -0.2818870000 -2.8232240000

H 2.6093020000 -1.9513290000 -2.9011200000

C 2.0549280000 -0.9049840000 -1.0882780000

O 4.3783320000 -3.9853010000 -1.1295130000

H 1.7916960000 -1.8328910000 -0.5697230000

H 2.5632830000 -0.2714880000 -0.3483140000

C 0.7637420000 -0.2158350000 -1.5064860000

H 0.9720710000 0.6291030000 -2.1691780000

H 0.1034530000 -0.9047240000 -2.0463480000

C 0.0886800000 0.2840760000 -0.2446010000

H -0.0998110000 -0.5278330000 0.4568440000

H 0.7226810000 1.0195050000 0.2588990000

N -1.2109650000 0.9326780000 -0.5307490000

H -1.2134630000 1.4314030000 -1.4374950000

H -1.4941310000 1.6068730000 0.1998730000

O 3.9183070000 -2.4225770000 0.4026680000

H 3.5577310000 -3.1912260000 0.9425920000

N 5.2721890000 -1.9404310000 -2.9029730000

H 6.2073410000 -2.2049340000 -2.5817540000

H 4.9038120000 -2.7188870000 -3.4465270000

H -1.9938150000 0.2668960000 -0.5923190000

O -3.7134950000 0.1281970000 -0.5346070000

H -4.1122700000 -0.7530480000 -0.4893530000

H -4.4251390000 0.6914600000 -0.8841270000

O 6.2430940000 -1.6462640000 1.7302380000

H 5.9798990000 -1.7292710000 2.6653970000

H 5.3903850000 -1.7245910000 1.2664520000

O 7.1170720000 -4.0101180000 0.4706330000

H 6.3139470000 -4.3471240000 0.0479930000

H 6.8087350000 -3.2259460000 0.9761360000

O 8.0209850000 -2.5572300000 -1.7457270000

H 7.7337170000 -3.1679030000 -1.0332130000

H 7.8935920000 -1.6664900000 -1.3447320000

O 2.8958040000 -4.5344360000 -3.6999330000

H 2.9381700000 -3.7096430000 -4.2391670000

H 3.3712710000 -4.3155010000 -2.8807010000

O 3.2106420000 -2.4638950000 -5.4833680000

H 2.9558210000 -1.5471210000 -5.2733160000

H 4.1717020000 -2.3987950000 -5.6806690000

O 5.3055930000 0.2744240000 -4.4266170000

H 4.3927090000 0.3933270000 -4.7731480000

H 5.2719110000 -0.5795070000 -3.8864040000

O 2.6930560000 0.3443910000 -5.2879340000

H 2.3006860000 0.9500290000 -4.6441950000

H 2.6041060000 0.7952520000 -6.1396680000

O 5.8786670000 -1.9067750000 -6.1602500000

H 5.7375260000 -1.0141110000 -5.7920590000

H 6.5603620000 -2.2687160000 -5.5518970000

O 8.1030370000 -2.3629290000 -4.5358400000

H 8.1791750000 -1.3856640000 -4.4966420000

H 8.0344900000 -2.6066460000 -3.5907860000

O 8.0501510000 0.4498610000 -4.3114340000

H 8.5335110000 0.8602110000 -5.0415960000

H 7.1145950000 0.4693460000 -4.5979100000

O 6.2858400000 1.6478390000 -2.2530030000

H 7.1535580000 1.6282500000 -2.6948480000

H 5.7305990000 1.1747450000 -2.9143830000

O 5.3460400000 3.9076990000 -3.7597660000

H 5.6459520000 3.2541860000 -3.0990460000

H 4.5705620000 4.3071130000 -3.3454880000

O 7.5358200000 -0.1981880000 -0.4520990000

H 6.9322140000 0.3603400000 -0.9819070000

H 7.0161390000 -0.5166910000 0.3078520000

O 5.3921800000 -4.9236430000 -5.0571320000

H 4.4950700000 -4.9199100000 -4.6730080000

H 5.9527870000 -4.9852680000 -4.2585040000

O 6.5652470000 -5.1797410000 -2.5323180000

H 5.8133260000 -4.6828300000 -2.1612880000

H 6.3071740000 -6.1034930000 -2.4051460000
